# Moral trade‐offs reveal foundational representations that predict unique variance in political attitudes

**DOI:** 10.1111/bjso.12781

**Published:** 2024-07-09

**Authors:** Amrita Ahluwalia‐McMeddes, Adam Moore, Calum Marr, Zara Kunders

**Affiliations:** ^1^ Division of Psychology University of Dundee Dundee UK; ^2^ Department of Psychology University of Edinburgh Edinburgh UK; ^3^ Centre for Public Health Queen's University Belfast Belfast UK

**Keywords:** drift‐diffusion, moral foundations theory, moral intuition, moral judgement, moral trade‐off

## Abstract

Moral Foundations Theory (MFT) explains variation in moral judgements on the basis of multiple innate, intuitive foundations and has been subject to criticism over recent years. Prior research has tended to rely on explicit self‐report in the Moral Foundations Questionnaire (MFQ). In contrast, we seek to capture intuitive choices between foundations in a novel task – the Moral Foundations Conflict Task (MFCT). Across four studies, responses on this task reflect foundations measured by the MFQ (study 1), are not altered under cognitive load or reduced cognitive control (studies 2a and 2b); and explain unique variance in political orientation and related constructs (study 3). Furthermore, using responses and response times generated on the MFCT, we present a computationally explicit model of foundation‐related intuitive judgements and show that these patterns are consistent with the theoretical claims of MFT. These findings show that the MFCT outperforms the MFQ and can contribute to the understanding of moral value conflicts, furthering debate on the nature of moral values.

## BACKGROUND

How do we judge if denying safety to vulnerable refugees is worse than overloading publicly funded services? There are two main threads in moral judgement research – one focused on the algorithms with which such judgements are made (Crockett, 2013; Cushman, 2013; Cushman & Greene, 2012; Greene et al., 2004; Moore, 2011; others) and another on the representations upon which algorithms operate (Gawronski et al., 2017; Gawronski & Beer, 2017; Greene et al., 2001, 2004; Haidt, 2001; Moore et al., 2008, 2011; Rai & Fiske, 2011; Shweder et al., 1997). In the latter, Moral Foundations Theory (MFT) proposes (at least) five primary representational types. MFT enjoys significant predictive utility, though it has also been criticized on several fronts. Here, we attempt to add to existing debates over MFT by developing a task that minimizes several extant criticisms and connects it more firmly to the algorithmic level by modelling choices as drift‐diffusion processes – decisions between the relative evidence produced by competing representations.

### Predictive success of MFT

MFT explains moral divergence as varying manifestations of multiple intuitive foundations (Haidt & Joseph, 2004, 2007) – care (concerns about protection, preventing suffering), fairness (reciprocity, justice), loyalty (social group membership), authority (obeying superiors, respecting traditions) and purity (physical and spiritual purity).^1^ These are representational templates that focus on aspects of moral problems, present early in development, require minimal social learning, trigger relatively automatically and explain variance in moral judgements (Graham, 2010; Graham et al., 2013; Haidt, 2001; Haidt & Graham, 2007; Haidt & Joseph, 2004).

MFT accounts for moral disagreement across individuals, with work on liberal‐conservative political orientation remaining the most widely replicated (Graham et al., 2013): liberals endorse care and fairness more strongly than authority, loyalty and purity, whereas conservatives value these relatively equally (Franks & Scherr, 2015; Graham et al., 2009, 2011, 2012; Milesi, 2016, 2017; Nilsson & Erlandsson, 2015; Van Leeuwen & Park, 2009). Endorsement patterns correlate with sociopolitical attitudes towards authoritarianism and social dominance orientation (Federico et al., 2013; Kugler et al., 2014; McAdams et al., 2008), prejudice (Van de Vyver et al., 2016), religious orientation (Bulbulia et al., 2013), charitable giving (Nilsson et al., 2016; O'Grady & Vandegrift, 2019), poverty (Low & Wui, 2016), sexism (Vecina & Chacon, 2019; Vecina & Pinuela, 2017), rape (Barnett & Hilz, 2018), homosexuality (Barnett et al., 2018) and climate change (Dawson & Tyson, 2012; Dickinson et al., 2016; Vainio & Mäkiniemi, 2016), as well as non‐political constructs, such as personality (Hirsh et al., 2010; Lewis & Bates, 2011) and attachment style (Koleva et al., 2014).

### Challenges to MFT

Notwithstanding this success, there are debates over MFT's structural basis and the number of distinct moral foundations (Jacobsin, 2008; Jost, 2012; Kugler et al., 2014; Schein & Gray, 2015, 2018). Critics argue that existing evidence does not robustly support five separate foundations. A two‐factor model can, in some cases, adequately explain the data (Van Leeuwen & Park, 2009; Wright & Baril, 2011; Yilmaz et al., 2016), and certain foundations might be better understood as aspects of broader ideological attitudes, such as authoritarianism and group‐based inequality (Federico et al., 2013; Hadarics & Kende, 2017; Kugler et al., 2014; McAdams et al., 2008; Milojev et al., 2014; Van Leeuwen & Park, 2009; Yilmaz & Saribay, 2017). Additionally, dyadic morality (Schein & Gray, 2015, 2018) and relational templates (Rai & Fiske, 2011, 2012) approaches suggest that what are discussed as distinct foundations might be contextually provoked variations of a simpler, more fundamental moral understanding (cf. Simpson et al., 2016).

Top‐down processes may also influence moral judgements, aligning intuitive moral responses with explicit identity‐related beliefs. This interaction is complex – some experiments report liberals becoming more conservative in their judgement patterns under cognitive load, though able to adjust when the conflict between intuitive and explicit identity‐related beliefs is obvious (Skitka et al., 2002), while others report conservatives becoming more like liberals (Wright & Baril, 2011). In both cases, the explanation offered is that load interferes with the top‐down alignment of intuitive responses with explicit beliefs. Other work fails to replicate these effects (Alper & Yilmaz, 2019; Graham et al., 2013; Isler et al., 2021) and there is evidence that load shifts patterns of responding regardless of political orientation/identity (Van Berkel et al., 2015; Yilmaz & Saribay, 2017), again possibly due to interfering with the top‐down alignment of intuitive responses. Graham (2010) proposes that intuitive‐level liberal‐conservative differences are much weaker than explicit‐level differences, suggesting that liberals are effortfully suppressing foundations more associated with conservative responding. Furthermore, recent studies suggest that the traditional model of moral foundations could be refined to reveal subcomponents within the foundations (Atari et al., 2022; Zakharin & Bates, 2021) and there is ongoing debate about the broader spectrum of moral values beyond the established framework (Curry, 2016; Curry et al., 2019). Thus, there is disagreement over how many foundations there are (two, five, or possibly seven) and whether they are distinct representations, merely contextual expressions of more fundamental underlying moral templates, or a more general worldview/political orientation linked to social identity (cf., Atari et al., 2022; Pedersen & Moore, 2023).

While dual‐task/cognitive load manipulations yield ambivalent results, others have probed moral intuitions using neurophysiological/neuroimaging techniques (Cannon et al., 2011; Graham, 2010; Hopp et al., 2023; Khoudary et al., 2022, study 5), manipulations of automatic foundation‐related triggers (Helzer & Pizarro, 2011; Horberg et al., 2009; Schnall, Benton, & Harvey, 2008; Schnall, Haidt, et al., 2008), evaluative priming (Graham, 2010, study 3), affect misattribution (Graham, 2010, study 4), implicit association tests (Schein & Gray, 2015, studies 4/7) and, of particular interest here, a speeded trade‐off task (Graham, 2010, study 2). However, interpreting the contributions of these studies to the outstanding questions just noted is complicated by replication failures in similar social priming paradigms (Doyen et al., 2012; Schimmack et al., 2017; Shanks et al., 2013), ambiguities in interpreting IAT associations (Andreychik & Gill, 2012; Uhlmann et al., 2006) and many of those studies' structural focus on only one foundation at a time, most often purity/degradation (Helzer & Pizarro, 2011; Horberg et al., 2009; Schnall, Benton, & Harvey, 2008; Schnall, Haidt, et al., 2008).

### Moral foundation trade‐offs

People respond differently to stimuli that more directly correspond to moral intuitions than to stimuli that do not (or do so to a lesser degree). Indeed, this is the core logic behind a significant portion of developmental research in this (Hamlin, 2013; Hamlin et al., 2011; preference for helping: Hamlin et al., 2007; Hamlin & Wynn, 2011; sensitivity to fairness violations: Sloane et al., 2012; preference for ingroup members: Hamlin et al., 2013) and other areas of cognition (e.g., numerosity: Feigenson & Carey, 2003; Xu et al., 2005; Xu & Spelke, 2000). Explicit trade‐offs involving morally valenced stimuli are predictive of attitudes to: whistleblowing (loyalty vs. fairness: Waytz et al., 2013); sexual outgroups (purity vs. care: Monroe & Plant, 2019); social justice protests (authority vs. fairness: Monroe et al., 2021); and Covid‐19 vaccination reluctance (purity/liberty vs. care: Ahluwalia‐McMeddes et al., 2024). However, trade‐offs are typically operationalized as differences in scores on the Moral Foundations Questionnaire (MFQ: Graham et al., 2011); (Ahluwalia‐McMeddes et al., 2024; Monroe & Plant, 2019, studies 1/2; Waytz et al., 2013, study 1), or as isolated foundations in trade‐offs against non‐moral goods, such as the amount of money required to endorse foundation violations (Graham et al., 2009, study 3; Monroe & Plant, 2019, study 3). Hence, this work does not entail participants directly choosing one foundation over another.

Graham (2010, study 2) used a task where participants chose which was worse in dyads of foundation violations – e.g., ‘Treating people unequally’ versus ‘Disobeying an authority’. However, this explored whether ideological differences persisted when participants answered with their first gut reaction (relative to when they deliberated) rather than to systematically explore evidence for/against a particular number of foundations. Here, we build on this trade‐off principle to measure intuitive foundation‐related choices as they dynamically compete with one another.

### The present research

Our primary goal is to generate data that inform debates over how many moral representations people intuitively use. To do this, we designed a task that reduces top‐down alignment of responding and thus better reflects bottom‐up intuitive strength, by using a wide variety of morally valenced stimuli and a speeded two‐alternative forced choice structure. In study 1, we test the extent of trade‐off responses in this task to reflect endorsements on the MFQ, as well as computationally model response times to evaluate how well the recovered parameters correspond to core claims from MFT. In studies 2a and 2b, we replicate the relationship between the trade‐off task and the MFQ and show that neither concurrent cognitive load nor calibrated alcohol intoxication alters the relationship with MFQ responding, thus indicating that performance on the task does not depend on top‐down alignment or reasoning/deliberation. In study 3, we explore if trade‐off preferences replicate well‐established relationships with political orientation and preferences for authoritarianism and group hierarchy.

## STUDY 1

To validate a new measure of moral intuitions, it must correlate with and differ from the established measure. We introduce the Moral Foundations Conflict Task (MFCT) to assess inter‐foundation trade‐offs and compare responding to MFQ self‐reports. This approach aims to contribute to the debate on the number of moral foundations, with MFT suggesting at least five distinct types underpin moral intuitions. Accordingly, MFCT responses and response times should exhibit five distinct patterns reflecting these underlying representations and their hierarchical preferences. Deviations from this pattern could support alternative theories (e.g., Rai & Fiske, 2011; Schein & Gray, 2015).

### Method

#### Participants

A G*power analysis (Faul et al., 2007, 2009), *a* = 0.05, *β* = .20, for a one‐tailed correlation (Pearson's *r* = .30) identified a sample size of 67. To allow for exclusions, we recruited a total of 78 UK students. Three participants were removed due to missing data, leaving a final sample of 75 (68% female, *M*_age_ = 24.09; *SD*_age_ = 4.48). Participation lasted 15–20 min, and participants were paid 3GBP. For studies reported here, ethical approval was granted by the university's ethics committee and participants' informed consent was obtained. Data were collected in May 2015.

To identify error trials on the task, trials with RTs <400 ms and >15 s were excluded (see Supporting Information for how cut‐offs were identified). Thirteen trials (<0.01%) were removed across 8 participants, leaving 11,987 trials (*M*_RT_ = 2542 ms; *SD*_RT_ = 1566 ms).

#### Measures and procedure

Participants completed two measures in randomized order in a lab setting.

##### Moral foundations questionnaire

We employed the widely‐used 30‐item Moral Foundations Questionnaire (MFQ‐30; Graham et al., 2011; *α*s: care = 0.38, fairness = 0.55, authority = 0.63, loyalty = 0.71, purity = 0.79, cf., Graham et al., 2011), developed to measure the first five foundations in the theoretical framework. Lower *a*s are common with the MFQ, arguably due to a balance between internal consistency and comprehensive coverage (Graham et al., 2011).

##### Moral foundations conflict task

This task was presented using E‐Prime 2.0 (Psychology Software Tools) and required speeded dichotomous choices between stimuli adapted from the Moral Foundations Dictionary (Graham et al., 2009) – see Supporting Information. The task is split into four blocks: (1) Virtue Active – active virtue behaviour; (2) Virtue Passive – passive virtue traits; (3) Vice Active – active vice behaviour; and (4) Vice Passive – passive vice traits (see Table 1). This structure redresses tendencies to focus on vices (Clifford et al., 2015; Garvey & Ford, 2014; Graham, 2010, study 2; Graham et al., 2009, study 3; Landy, 2016; Royzman et al., 2014; Schein & Gray, 2015) and on either action (Graham, 2010; Graham et al., 2009) or passive traits (Graham, 2010, study 3). Overall responding remained stable across formulations and is globally analysable (Ahluwalia, 2020).

**TABLE 1:** Example items in MFCT.

Block	Prompt	Sample items
Virtue Active	It is better to	Treat everyone equally (*Fairness*) Put family before yourself (*Loyalty*) Protect defenceless animals (*Care*)
Virtue Passive	It is better to be	Respectful (*Authority*) Pious (*Purity*) Compassionate (*Care*)
Vice Active	It is worse to	Do something disgusting (*Purity*) Act in an obstructive manner (*Authority*) Cheat to get ahead (*Fairness*)
Vice Passive	It is worse to be	Cruel (*Care*) Promiscuous (*Purity*) Selfish (*Loyalty*)

*Note*: For each item, the corresponding foundation is provided in parentheses. A total of 75 different words/phrases were included in the task, matched for valence and lexical characteristics (see Supporting Information).

Participants randomly started with either both virtue or vice blocks and within these, with either active or passive items. Participants were instructed to make choices as quickly as possible, based on gut responses. There were a total of 14 practices (7 virtue, 7 vice) and 160 test trials (16 for each inter‐foundation combination, 4 pairings per combination per block). Items appeared randomly on the left/right. We recorded trial responses (which stimulus was chosen) and RTs (in ms).

#### Analytic approach

Analyses were conducted in R (v3.6.1) (R Core Team, 2019), except for the Bayesian hierarchical drift‐diffusion model fit using Python (v3.1.7) and the hddm package (v0.9.7; Wieki et al., 2013). The MFCT produces two kinds of data – responses and RTs. Here, we present drift‐diffusion modelling of RTs, with related NHSTs reported in Supporting Information (showing small RT increases when foundations were more closely valued). We apply two kinds of correlation coefficients to compare MFQ/MFCT responding: Pearson's *r* for between‐participant foundation scores; and bootstrapped Kendall's *τ* for within‐participant match between ordered preferences of foundations (bootstrapped using *boot* package: v1.3‐24; Canty & Ripley, 2019). We take mean Kendall's *τ*s to indicate the overall extent to which preferences on the MFCT correspond to those on the MFQ. We follow Cohen (1988), with *r* = .10, *r* = .30 and *r* = .50 interpreted as small, medium and large, respectively.

### Results

#### Responses

MFCT response scores were created for foundations based on the presentation choice proportion (0 = never chosen, to 1 = always chosen). Mean foundation scores are shown in Table 2 along with Pearson's *r* correlations. Correlations were positive and (approaching) large for care, loyalty and purity, though not significant for authority or fairness. Kendall's *τ* correlations ranged from −.89 to 1.00, with a mean of *τ*(75) = .50. Bootstrap resampling gave an estimate of *τ*_Boot_ = .51 (*SE* = 0.05, 95% CI = [0.43, 0.61]), indicating a 50% match between the ordering of foundations measured by the MFCT and MFQ.

**TABLE 2:** Descriptive statistics and Pearson correlations for study 1 variables.

	*M*	*SD*	1	2	3	4	5	6	7	8	9
1. Care‐MFQ	3.9	0.5									
2. Fairness‐MFQ	3.9	0.5	.25								
3. Authority‐MFQ	2.4	0.8	−.09	−.06							
4. Loyalty‐MFQ	2.5	0.8	−.01	−.02	.54***						
5. Purity‐MFQ	2.0	1.0	.01	.00	.49***	.38**					
6. Care‐MFCT	0.70	0.13	.47***	.14	−.33*	−.42**	−.30^†^				
7. Fairness‐MFCT	0.57	0.13	−.06	**.20**	−.28	−.45***	−.41**	.18			
8. Authority‐MFCT	0.37	0.11	−.23	.00	**.30** ^**†**^	.26	.17	−.53***	−.31^†^		
9. Loyalty‐MFCT	0.46	0.13	.01	−.13	.17	.55***	.04	−.35*	−.39**	−.10	
10. Purity‐MFCT	0.39	0.13	−.22	−.21	.19	.09	.53***	−.38**	−.52***	.11	−.20

*Note*: ^†^ *p* < .10, *p < .05, **p < .01, ***p < .001. MFQ scores are on 0 to 5 scale, MFCT scores are on 0 to 1 scale. *p*‐values corrected for multiple comparisons (Bonferroni). Correlations between the same foundations on the MFQ and on the MFCT have been highlighted in bold.

#### Response times

Sequential sampling models have several advantages over NHSTs, including the ability to model speed‐accuracy trade‐offs, trial‐by‐trial and condition‐based adjustments in caution or bias and parameters that more cleanly correspond to underlying theoretical constructs of interest (Forstmann et al., 2016; Ratcliff & Smith, 2004; Smith & Ratcliff, 2004) – in this case, the strength of representations driving responding. Based on signal detection theory, such models assume that when presented with two options, decision‐makers sample information associated with each, accumulating that information until the asymmetry (i.e., the balance of information favouring one option) passes some threshold which triggers a decision. In our case, the rate of information accumulation, or drift rate, should reflect the strengths (availability or quality of information) of the underlying foundations. Assuming thresholds for making a decision remain relatively stable over trials (i.e., do not interact with foundation), drift rate will be a function of *both* combined overall strengths of the relevant representations (stronger representations provide more information more quickly and thresholds are reached sooner) and asymmetry in the relative strengths (more evenly matched foundations will require more information accumulation before clearly favouring one over the other).

There are three key outcomes for this analysis. MFT requires a pattern of drift‐rates (evidence accumulation) that (1) reliably discriminate foundations from one another (there will be five distinguishable drift‐rates rather than all foundations yielding comparable results or being indistinguishable from each other) (2) in a way that respects the absolute strength of underlying representations (must be ordered such that more valued foundations yield higher drift‐rates overall) as well as (3) their relative strengths (pairing closely‐valued foundations together should yield lower rates of evidence accumulation, compared to more asymmetrical trials).^2^ Failure to find this pattern would count against MFT in its current form. Failure to fit a model with these properties that can explain meaningful variance in *both* responses and RTs would also undermine any contribution from the MFCT.

##### Drift‐diffusion model

For each participant, the foundation rated most highly on the MFQ (as the more established measure and to prevent ‘double‐dipping’ in the data) was treated as most valued, second highest as second most valued, etc. Across trials, responses were coded as correct when the participant chose the more valued option and incorrect otherwise. We then fit a Bayesian hierarchical drift‐diffusion model to estimate three parameters: response caution, non‐decision time and drift rate. It is beyond the scope of the current work to explore all possible parameterisations of such a model; thus, here we report the statistically best‐fitting model tested which is also compatible with the hypotheses above. Because ties in MFQ scores between two (or more) foundations make assigning correct/incorrect trials impossible, we dropped such trials (<5% of trials). Response caution (*a*) was a joint function of both valence (Virtue vs. Vice) and stimulus type (Active vs. Passive), capturing the differential levels of caution exhibited by participants when judging these 4 categories of stimuli. Non‐decision response time (*t*) was estimated as a function of stimulus type, reflecting differing verbal complexity. Response bias (*z*) was fixed at 0 (reflecting the fully counterbalanced nature of stimuli x condition x keyboard mapping and within‐participant randomisation of presentation).

Of primary interest, the rate of evidence accumulation (drift rate, *v*) was modelled as an additive function of both valence and each trial's unique pairwise rank combination (e.g., 1st foundation vs. 2nd, 2nd vs. 3rd, etc.), given that model testing indicated no interaction between these factors for drift rate. All hypothesis tests reported below are Bayesian tests of credibility and are reported as probability (*p*) of posterior estimate inequalities by condition.

##### Response caution

**FIGURE 1:**
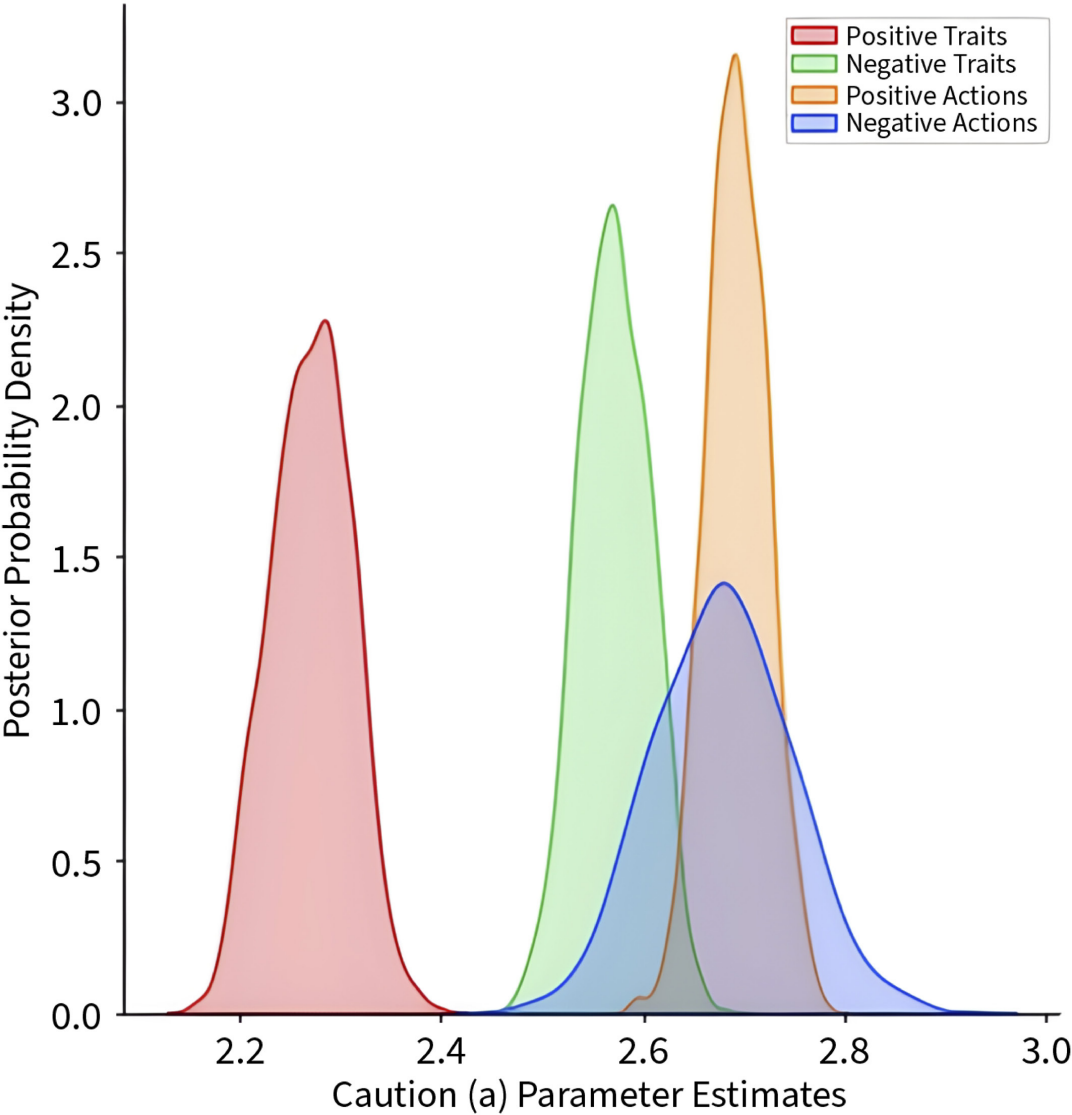
Posterior density estimates of the caution (a) parameter as a function of Valence (Positive vs. Negative) and Stimulus Type (Action/Active vs. Trait/Passive). *Note*: Estimates are intercept adjusted to share a common positive scale.

Response caution (*a*) varied as a function of both valence and stimulus type (Figure 1), with negative traits resulting in more caution than positive traits, *p*(*a*‐Vice_Passive_ > *a*‐Virtue_Passive_) = 1.0 but no credible difference between positive and negative actions, *p*(*a*‐Vice_Active_ > *a*‐Virtue_Active_) = .33.

#### Non‐decision time

Non‐decision (i.e., reading) time (*t*) credibly differed as a function of stimulus type (Active, *M* = 1.00 s, *SD* = 0.03 s, vs. Passive, *M* = 0.79 s, *SD* = 0.03 s), *p*(*t*‐Active > *t*‐Passive) = 1.0. This likely reflects greater average complexity of active versus passive stimuli.

##### Drift rate

**FIGURE 2:**
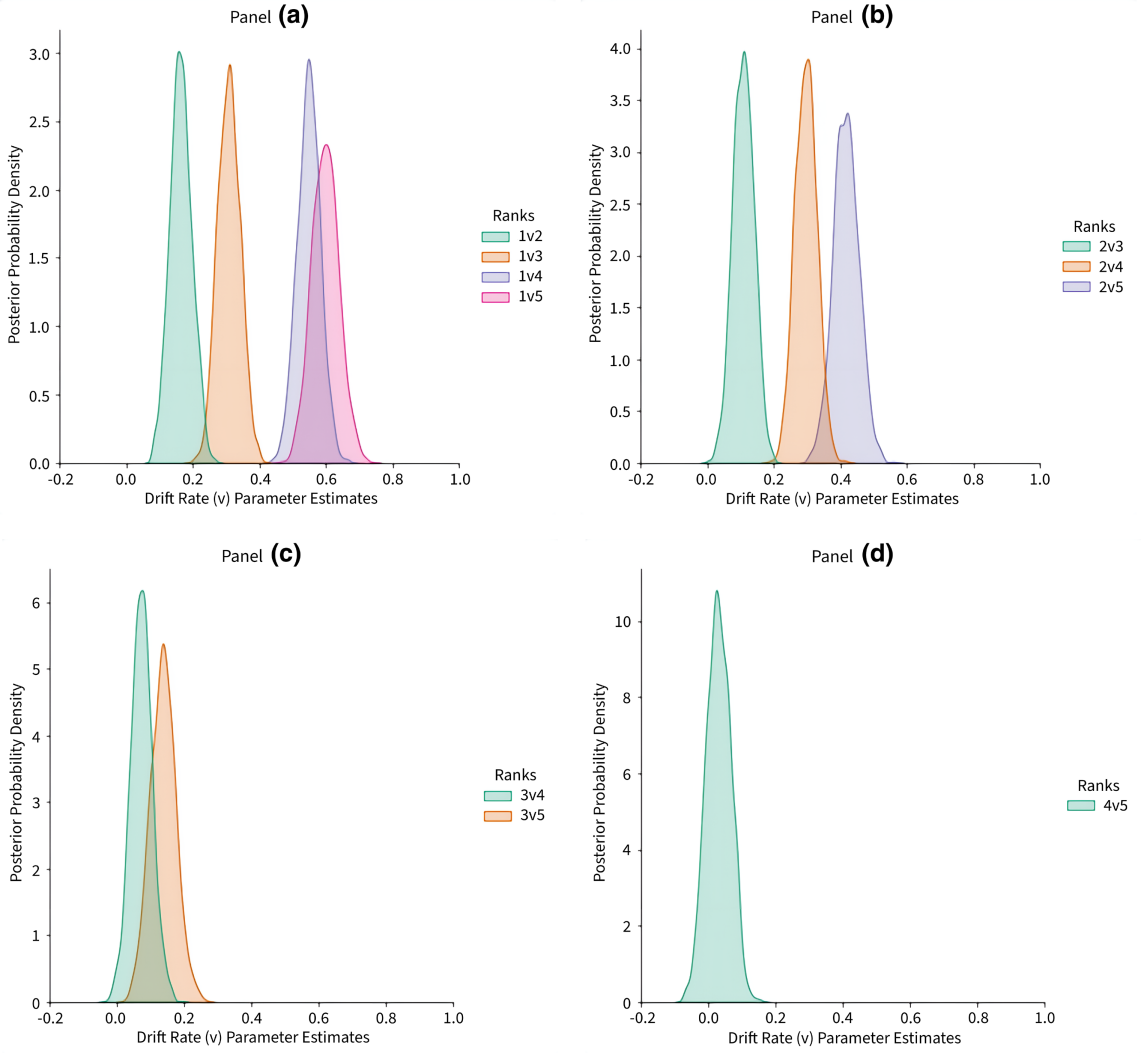
Bayesian posterior density estimates of the drift‐rate (*v*) parameter as a function of unique moral foundation ranking and pairings. *Note*: Estimates are intercept adjusted to share a common *X*‐axis. Panel a displays the moral foundation chosen most often (1) pitted against all other foundations in order of their preference (2–5). Panel b displays foundation chosen second most often (2) pitted against all other foundations (3–5). Panel c displays foundation chosen third most frequently pitted against (4–5). Panel d displays foundation (4) pitted against (5). Note that *X*‐axes are consistent across panels (for comparison), but *Y*‐axes are not.

Our primary hypotheses concerned drift rate (*v*): more valued foundations should produce larger absolute drift‐rates and more closely matched foundations should result in lower drift rates. As with response caution, there is a negativity bias with evidence accumulating faster for negative versus positive stimuli, *p*(*v*‐Vice > *v*‐Virtue) = 1.0. Examining drift rate as a function of rank ordering (i.e., 1st through 5th) and ranks apart reveals a regular pattern conforming to our expectations (Figure 2, panels a–d).

As shown in Figure 2, drift rates clearly discriminate foundation pairings in a way that respects both the absolute and relative strengths of underlying representations. In the absolute case (comparing same‐coloured distributions across panels), pairings that are equivalently spaced (i.e., one rank‐apart) nevertheless differ depending on the absolute strength of the foundations on offer. Comparing 1v2 (Panel A, green) to 2v3 (Panel B, green) demonstrates a credibly higher drift rate, *p*(*v‐*1v2 > *v‐*2v3) = .95, indicating faster evidence accumulation. Similar comparisons between 2v3 versus 3v4 and 3v4 versus 4v5 yield results trending strongly in the same direction (*p*s ≥ .85). Comparing pairings two and three ranks‐apart yields largely similar results: 1v3 versus 2v4 do not differ, *p* = .63, but 2v4 versus 3v5 and 1v4 versus 2v5 do, all *p*s = 1.0.

In the relative case, similarly valued foundations result in lower drift‐rates because the evidence accumulating is ambivalent for determining a correct response. Thus, more time is required to sample enough information to cross the decision threshold for a given level of response caution. As pairing ranks become more divergent, evidence in favour of one option becomes clearer, reflected in credibly higher drift‐rates and faster decision times. This pattern repeats across all relevant combinations within each panel; moving rightward across the X‐axis, all pairwise combinations of these within‐participant estimates in panels A–C are credibly different (all *p*s ≥ .94), with the difference between 1v4 and 1v5 (Panel A) trending strongly in the same direction (*p* = .87).

To evaluate model fit and determine if this approach explains enough variance in our data to warrant strong inferences, we computed posterior predictive checks. Fitted model parameters were used to simulate multiple datasets, then means from these simulations were compared to the empirical data (Table 3). The model is extremely accurate in reproducing the observed response and RT data; in the latter case, it accurately reproduces observed means generally and within every RT quintile.

**TABLE 3:** Posterior predictive checks of drift‐diffusion model accuracy.

Statistic	Mean (data)	Mean (Sim)	SD (Sim)	SEM	MSE	Credible	Posterior quantile	Mahalanobis distance
Accuracy	0.64	0.65	0.11	<0.001	0.01	TRUE	42.39	0.12
Mean U Bound	2.46	2.52	0.86	<0.01	0.74	TRUE	54.86	0.07
SD U Bound	1.50	1.29	0.62	0.04	0.43	TRUE	70.35	0.53
10% U Bound	1.14	1.36	0.41	<0.05	0.22	TRUE	35.42	0.53
30% U Bound	1.56	1.71	0.54	0.02	0.31	TRUE	45.87	0.28
50% U Bound	2.04	2.14	0.71	0.01	0.52	TRUE	51.33	0.14
70% U Bound	2.72	2.79	0.99	<0.01	0.98	TRUE	55.13	0.07
90% U Bound	4.35	4.15	1.62	0.04	2.65	TRUE	63.32	0.12
Mean L Bound	−2.66	−2.57	0.92	<0.01	0.85	TRUE	38.50	0.09
SD L Bound	1.68	1.29	0.68	0.16	0.62	TRUE	76.95	0.58
10% L Bound	1.18	1.40	0.44	0.04	0.24	TRUE	38.03	0.48
30% L Bound	1.65	1.75	0.58	0.01	0.35	TRUE	50.24	0.18
50% L Bound	2.15	2.19	0.78	<0.01	0.61	TRUE	55.63	0.06
70% L Bound	2.94	2.85	1.09	<0.01	1.19	TRUE	61.82	0.09
90% L Bound	4.82	4.17	1.75	0.42	3.50	TRUE	71.24	0.37

*Note*: U = Upper; L = Lower; Sim = simulated datasets; SD = standard deviation of the mean of the simulated datasets; SEM = standard error of the mean of those simulations; MSE = mean squared error of the simulated means from the empirical means; Credible refers to whether or not the empirical mean falls within the 95% credible interval around the mean of the simulations; Quantile indicates the specific region of the 95% credible interval in which the corresponding empirical mean falls.

### Discussion

Foundation preferences emerging from the MFCT correlated at *τ*(75) = .50 with self‐reported moral values on the MFQ. This magnitude is perhaps unsurprising – choices on the MFCT are, in a sense, explicit, but because the task includes many such judgements, in unpredictable pairings, framed in multiple ways and requiring quick (but accurate) responding, it is less plausible that responses are due to participants' conscious decisions to align their choices with self‐presentational/ideological concerns. Rather, we interpret preferences as cleaner indicators of bottom‐up intuitive foundation strength. Hence, there is a moderate but imperfect match between conflict‐revealed foundation preferences and explicitly‐reported foundation endorsement.

RT modelling yields several novel findings that coincide with this interpretation. The model discriminates between foundation pairings in a way that suggests multiple distinct underlying representations ordered in reliable ways that conform to MFT. This explains an otherwise counterintuitive result – decision times for trade‐offs that involve core moral values (and therefore ones we might assume a priori to be *harder* moral decisions) are often faster than those involving comparatively non‐valued foundations. Here, the ‘harder’ moral choices involve representations that produce higher rates of evidence accumulation. This echoes recent RT modelling work that pits action‐oriented/deontological considerations against outcome‐oriented/utilitarian ones (Baron et al., 2012; Baron & Gürçay, 2017). The problems that take the longest to respond to are those in which the two considerations are equivalently strong for a given participant. Our results are similar – the decisions with the lowest drift‐rates are those that involve equivalently‐paired representations, even when these are the strongest/most valued or the weakest/least valued.

The drift‐diffusion approach here goes one step further than previous work by linking MFT to precise, neurally plausible, computationally explicit decision processes that instantiate clear roles for foundation representations. Similar work has been fruitful in studying single foundation judgements (e.g., fairness: Andrejević et al., 2020, 2022; sharing/cooperation: Gallotti & Grujić, 2019; Hutcherson et al., 2015; Krajbich et al., 2015; harm/punishment: Pärnamets et al., 2015; Son et al., 2019; Yu et al., 2021). Additionally, neuroimaging work (e.g., Hopp et al., 2023; Khoudary et al., 2022) provides evidence for dissociable neural foundation representations, even where behavioural signatures are indistinguishable. Ultimately, this suggests that the representations underpinning moral intuitions can be delineated by both the MFQ and the MFCT, but that the latter may possess structural properties that give a clearer measurement of foundation representations and their relative strength.

However, while implausible, the MFCT might track not intuitive responses, but only faster versions of the same deliberated judgements as the MFQ. Studies 2a and 2b test this with the addition of a cognitive load/control manipulation to the MFCT.

## STUDIES 2A AND 2B

We argue that the MFCT diminishes top‐down influence due to speeded choices between numerous and diverse stimuli, making it challenging for participants to align their responses based on explicit beliefs. We next test the impact of increased cognitive load (study 2a) and reduced cognitive control (study 2b) on the MFQ–MFCT relationship. We anticipate that the association will be unaffected by these manipulations, maintaining the stability of the MFQ–MFCT correlation. Conversely, if MFCT responses are based on deliberated judgements, these interventions may weaken the MFQ–MFCT correlation due to more random responses or less consistency across choices. We also expect to replicate the MFQ–MFCT response correspondence from study 1.

We discuss both together, below.

### Study 2a

#### Method

##### Participants

A G*power (Faul et al., 2007, 2009) analysis, *a* = 0.05, *β* = .20, for a medium effect size (Cohen's *q* = .50) for a one‐tailed test of difference between two independent correlations yielded *N* = 100. Participants were UK students, paid 3GBP and randomly assigned to either the control or the load condition. One participant was removed due to missing data, leaving a final sample of 99 participants (79% females, *M*_age_ = 22.79; *SD*_age_ = 3.13), with 50 in the control and 49 in the load‐condition. Data were collected in April/May 2016.

##### Measures and procedure

Participants completed MFQ and MFCT in randomized order in the lab. Control participants followed the same procedure as study 1.

###### Cognitive load manipulation

The load manipulation imposed concurrent tone‐counting (see Skitka et al., 2002). Throughout each block, load‐condition participants heard a randomly high (40%) or low (60%) pitched tone every 2 seconds through headphones. Tone sequence varied every block. Participants were instructed to count only the number of high‐pitched tones at the same time as completing the MFCT. After each block, participants reported the number of high‐pitched tones. Upon completion, participants indicated perceived performance and difficulty (see Supporting Information).

#### Results

Here we focus only on the overall relationship between the rank‐ordering of foundations on the MFCT and MFQ scores and whether it is modulated by load (see Supporting Information for additional analyses). We replicated the overall MFQ–MFCT correlation, mean Kendall's *τ*(99) = .56, *τ*_Boot_ = .58 (*SE* = 0.04, 95% CI = [0.52, 0.67]).

There was no evidence that correlations differed as a function of cognitive load, *τ*_BootLOAD_ = .65 (*SE* = 0.05, 95% CI = [0.61, 0.76]), versus control, *τ*_BootCONTROL_ = .60 (*SE* = 0.05, 95% CI = [0.56, 0.71]), providing moderate evidence for the null‐hypothesis, Mann–Whitney *U* = 1100.00, *Z* = −0.88, *p* = .40, *r* = .09, BF_10_ = 0.26 (cf., Jeffreys, 1939/1961; Lee & Wagenmakers, 2014; van Doorn et al., 2020).

Tone‐counting performance was generally poor – out of approximately 20 high tones heard per block, participants tended to miscount tones by a mean of 2.77 (*SD* = 1.41), with around 35% making errors of 5 or more in at least one block.

### Study 2b

#### Method

##### Participants

Sample size was determined as in study 2a. A UK student sample of 90 participants was collected across two concurrent recruitment drives to control and alcohol conditions. We recruited to condition due to ethical considerations requiring participants in the alcohol‐condition to satisfy additional criteria (drawn from Bregu et al., 2017), including having experience with and no health conditions that precluded, alcohol consumption – see Supporting Information for self‐reported alcohol habits. Control and alcohol groups were compensated 3GBP and 5.50GBP, respectively, to reflect respective time entailed (20 and 40 min). Following previous criteria, one participant was removed. Final sample comprised 89 participants (64% females, *M*_age_ = 23.76; *SD*_age_ = 4.53), with 43 in the control and 46 in the alcohol‐condition. Data were collected in May/June 2017.

##### Measures and procedure

Participants completed the MFQ online up to one week before completing the MFCT in the lab. All other details for the control conditions are as previous. MFQ subscale reliabilities were acceptable (*a*s: care = 0.77; fairness = 0.73; authority = 0.72; loyalty = 0.73; purity = 0.83).

##### Cognitive control manipulation

Alcohol doses effectively reduce executive control (Bartholow et al., 2006; Bregu et al., 2017; Van Berkel et al., 2015). ‘Load’ condition participants were given a body‐weight calibrated alcohol dose before completing the MFCT, calculated to raise blood alcohol content (BAC) to 0.03, where attention and judgement begin to diminish (Dubowski, 1980) – see Supporting Information. Alcohol doses were consumed on arrival, followed by a 20 min period to allow absorption. Participants then completed the MFCT.

#### Results

Overall correlation between MFQ and MFCT remained as before, mean Kendall's *τ*(89) = .61, *τ*_Boot_ = .64 (*SE* = 0.03, 95% CI = [0.60, 0.74]). As above, correlations did not differ as a function of reduced cognitive control in the alcohol condition, *τ*_BootLOAD_ = .64 (*SE* = 0.04, 95% CI = [0.56, 0.71]), versus control condition, *τ*_BootCONTROL_ = .56 (*SE* = 0.05, 95% CI = [0.43, 0.65]), hence providing additional support for the null‐hypothesis, Mann–Whitney *U* = 886.00, *Z* = −0.85, *p* = .40, *r* = .09, BF_10_ = 0.38 (cf., Jeffreys, 1939/1961; Lee & Wagenmakers, 2014; van Doorn et al., 2020).

### Discussion

MFCT responses remained unaffected by cognitive load or alcohol, indicating that evaluating moral foundations does not rely on top‐down executive functions or reflective scrutiny. However, there were limitations, such as potential neglect of the tone‐counting task and unmeasured (achieved) blood alcohol concentration (BAC). Nevertheless, the results suggest that MFCT responses are resilient to attention and executive resource manipulations, supporting the notion that choices in the task are predominantly intuitive.

We now turn to assessing the MFCT's association strength with correlates such as political orientation and related constructs, in comparison to the MFQ. If the MFQ captures both foundation strength and political identity, it should correlate substantively with political identity measures. Since the MFCT is a different measure, its comparative effectiveness in predicting political orientation and related constructs will be a stringent test of its predictive power and the theoretical link between moral foundations and these constructs.

## STUDY 3

Study 1 found the MFCT corresponds with self‐reported MFQ scores but shows unique patterns in choices and RTs, suggesting novel quantifiable evidence of distinct moral foundations (cf., Andrejević et al., 2020; Baron et al., 2012; Baron & Gürçay, 2017). The MFCT might better reflect intuitive representations with reduced input from reflection or ideology, as supported by studies 2a and 2b. We now connect MFCT preferences to political orientation, right‐wing authoritarianism (RWA) and social dominance orientation (SDO), all known to correlate with MFQ scores.

Ideological variances in moral foundation endorsement are well‐documented (Franks & Scherr, 2015; Graham et al., 2009/2012; Davis et al., 2016) and linked to RWA and SDO. RWA is associated with a preference for authority and tradition (Altemeyer, 1996, 1998), while SDO reflects a preference for hierarchical social structures (Pratto et al., 1994; Sidanius & Pratto, 1999). Both are related to stronger support for loyalty, authority and purity, with SDO also linked to lesser concern for care and fairness (Federico et al., 2013; Hadarics & Kende, 2017; Kugler et al., 2014; McAdams et al., 2008; Milojev et al., 2014). RWA and SDO partially mediate the link between political orientation and moral foundations (Kugler et al., 2014), aligning with evidence that orientation/worldview may precede moral values (Hatemi et al., 2019; Strupp‐Levitsky et al., 2020). Do the intricate connections between moral intuitions and personality traits related to political ideology change when assessed via the MFCT?

We preregistered (https://osf.io/nbwz4) predictions of a positive correlation between the MFCT and MFQ of similar magnitude to that observed in the previous studies. We also predicted a replication of associations in the literature for both the MFCT and MFQ. We did not have specific predictions about how MFQ and MFCT models would compare. As with study 1, basic NHST analyses of RT are included in Supporting Information.^3^

### Methods

#### Participants

We preregistered sample size and exclusion criteria. A general UK sample of 854 participants were recruited using Prolific (www.prolific.co) and compensated 1.85GBP. Following preregistered criteria, 90 participants were removed due to missing data: 32 failed attention/compliance checks and 32 had >10% of trials excluded. The final sample consisted of 700 participants (61% females; *M*_age_ = 37.50; *SD*_age_ = 12.06). Data were collected in April 2018.

#### Measures and procedure

Participants completed five main measures in randomized order in an online study implemented in jsPsych (de Leeuw, 2015), including MFQ and MFCT. Subscale reliabilities for the MFQ were *α*s: care = 0.62, fairness = 0.65, authority = 0.79, loyalty = 0.73, purity = 0.81.

We administered three items by Carney et al. (2008) to measure political orientation.^4^ Responses were provided on a 7‐point scale ranging from 1 (Very liberal) to 7 (Very conservative), with a highly reliable index (*α* = .93). We used the 16‐item SDO scale (SDO‐6; Pratto et al., 1994; scaled from 1 to 7; *α* = .94) and the 8‐item RWA scale (Sibley & Duckitt, 2009; scaled from 1 to 7; *α* = .80).

### Results

#### Preregistered

Correlations between MFQ and MFCT scores were medium or stronger for all foundations (Table 4). Kendall's *τ* coefficients ranged from −1.00 to 1.00, with a mean of *r*_*τ*_(700) = .47, *r*_*τBoot*_ = .46 (*SE r*_*τBoot*_ = 0.01, 95% CI = [0.43, 0.49]). Authority, loyalty and purity positively correlated with conservatism, SDO and RWA. Care and fairness negatively correlated with SDO and conservatism. In addition, RWA negatively correlated with care and fairness more so on the MFCT, than on the MFQ.

**TABLE 4:** Descriptive statistics and Pearson correlations for study 3 variables.

	*M*	*SD*	1	2	3	4	5	6	7	8	9	10	11	12
1. Political Orientation	3.3	1.4												
2. SDO	2.4	1.1	.52***											
3. RWA	2.7	1.1	.50***	.45***										
4. Care‐MFQ	4.0	0.6	−.24***	−.42***	−.11*									
5. Fairness‐MFQ	3.8	0.6	−.30***	−.54***	−.19***	.56***								
6. Authority‐MFQ	2.9	1.0	.50***	.43***	.66***	.02	−.04							
7. Loyalty‐MFQ	2.7	0.9	.36***	.30***	.50***	.12*	.04	.69***						
8. Purity‐MFQ	2.4	1.1	.36***	.30***	.69***	.16***	.07	.70***	.60***					
9. Care‐MFCT	0.75	0.13	−.24***	−.33***	−.37***	.37***	.22***	−.34***	−.27***	−.28***				
10. Fairness‐MFCT	0.50	0.15	−.47***	−.49***	−.55***	.15***	.35***	−.52***	−.39***	−.47***	.20***			
11. Authority‐MFCT	0.42	0.13	.39***	.39***	.52***	−.21***	−.23***	.59***	.33***	.41***	−.57***	−.56***		
12. Loyalty‐MFCT	0.43	0.10	.22***	.29***	.19***	−.22***	−.25***	.19***	.32***	.10	−.36***	−.42***	.10^†^	
13. Purity‐MFCT	0.40	0.12	.26***	.32***	.37***	−.18***	−.23***	.25***	.17***	.38***	−.43***	−.55***	.17***	−.03

*Note*: ^†^ *p* < .10, *p < .05, ***p < .001. Political orientation, SDO and RWA, are on 1 to 7 scale. MFQ scores are on 0 to 5 scale, MFCT scores are on 0 to 1 scale. *p*‐values corrected for multiple comparisons (Bonferroni). Correlations between the same foundations on the MFQ and on the MFCT have been highlighted in bold.

##### Mediation models

We built two sets of path analyses (saturated and trimmed models) to test whether associations between political orientation and foundations on (a) the MFQ and (b) the MFCT, are mediated by SDO and RWA. Models for (a) are a direct replication of Kugler et al. (2014). For brevity, we report trimmed models here with saturated models in Supporting Information. Trimmed models excluded non‐significant pathways between SDO/RWA and foundations. Bootstrapped CIs were estimated for indirect paths (Kugler et al., 2014; Preacher & Hayes, 2008; Shrout & Bolger, 2002). Models were fit with the *lavaan* package (v0.6‐5; Rosseel, 2012).

##### MFQ model

**FIGURE 3:**
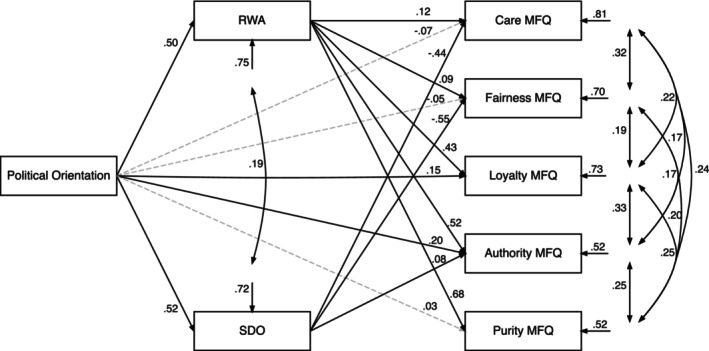
Trimmed path model fit with MFQ foundation scores. *Note*: Trimmed path model showing SDO and RWA mediate the relationship between political orientation and foundations on the MFQ. Path coefficients are standardized regression coefficients of the trimmed model. Broken lines indicate non‐significant paths at *p* > .05.

The trimmed model (Figure 3; also Table 5) fit well, TLI = 0.99 and RMSEA = 0.04 (Kugler et al., 2014: RMSEA = 0.02), yielding positive associations between political conservatism and SDO/RWA. Conservatism had direct positive paths to loyalty and authority. We partially replicate previous work (Kugler et al., 2014) with paths from political orientation to care, fairness and authority mediated by SDO, though we did not find an indirect path to loyalty. Similarly, we replicated indirect paths with RWA for the binding foundations. However, unlike Kugler et al. (2014), we found that paths from political orientation to care/fairness were positively mediated by RWA.

**TABLE 5:** Direct and indirect paths from political orientation to foundations on the MFQ.

	Care		Fairness		Loyalty		Authority		Purity	
	*β*	95% CI	*β*	95% CI	*β*	95% CI	*β*	95% CI	*β*	95% CI
Direct paths										
PO → MF	−.07	[−0.16, 0.02]	−.06	[−0.13, 0.03]	.15***	[0.07, 0.23]	.20***	[0.13, 0.27]	.03	[−0.04, 0.09]
SDO → MF	−.44***	[−0.52, −0.35]	−.55***	[−0.63, −0.48]	–	–	.09**	[0.03, 0.14]	–	–
RWA → MF	.12*	[0.03, 0.21]	.09*	[0.00, 0.18]	.43***	[0.33, 0.52]	.52***	[0.45, 0.59]	.68***	[0.62, 0.74]
Indirect paths										
PO → SDO → MF	−.23***	[−0.28, −0.18]	−.29***	[−0.35, −0.23]	–	–	.04**	[0.01, 0.07]	–	–
PO → RWA → MF	.06*	[0.01, 0.10]	.04^†^	[0.00, 0.09]	.21***	[0.16, 0.26]	.26***	[0.22, 0.30]	.34***	[0.29, 0.39]
*R* ^2^	.19		.30		.27		.48		.48	

*Note*: ^†^ *p* < .10, *p < .05, **p < .01, ***p < .001. *R* ^2^ signifies the proportion of variance in foundations explained by the trimmed model. Bootstrapped 95% confidence intervals with 5000 samples.

Abbreviations: MF, Moral Foundations; PO, Political Orientation.

##### MFCT model

**FIGURE 4:**
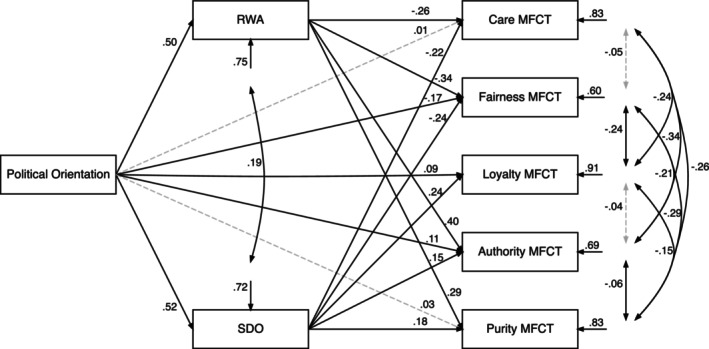
Trimmed path model fit with MFCT foundation scores. *Note*. Trimmed path model showing SDO and RWA mediate the relationship between political orientation and foundations on the MFCT. Path coefficients are standardized regression coefficients of the trimmed model. Broken lines indicate non‐significant paths at *p* > .05.

The trimmed MFCT model provided a better fit, TLI = 1.00 and RMSEA = 0.03 (Figure 4; also Table 6) with more theoretically compatible results, including positive associations between political orientation and SDO and RWA, as well as direct paths from political orientation to fairness, authority and loyalty. RWA mediated paths to authority and purity, but not loyalty. RWA also more strongly mediated negative indirect paths to care and fairness than in the MFQ model, where the indirect paths were counter‐intuitively positive.

**TABLE 6:** Direct and indirect paths from political orientation to foundations on the MFCT.

	Care		Fairness		Loyalty		Authority		Purity	
	*β*	95% CI	*β*	95% CI	*β*	95% CI	*β*	95% CI	*β*	95% CI
Direct paths										
PO → MF	.01	[−0.08, 0.10]	−.17***	[−0.25, −0.10]	.09*	[0.00, 0.17]	.11**	[0.03, 0.19]	.03	[−0.06, 0.11]
RWA → MF	−.26***	[−0.33, −0.18]	−.34***	[−0.40, −0.28]	–	–	.40***	[0.33, 0.47]	.29***	[0.20, 0.37]
SDO → MF	−.22***	[−0.31, −0.13]	−.24***	[−0.32, −0.17]	.24***	[0.16, 0.34]	.15***	[0.08, 0.23]	.18***	[0.09, 0.26]
Indirect paths										
PO → RWA → MF	−.13***	[−0.17, −0.09]	−.17***	[−0.21, −0.13]	–	–	.20***	[0.16, 0.24]	.14***	[0.10, 0.19]
PO → SDO → MF	−.12***	[−0.16, −0.07]	−.13***	[−0.17, −0.09]	.13***	[0.08, 0.18]	.08***	[0.04, 0.12]	.09***	[0.05, 0.14]
*R* ^2^	.16		.39		.09		.31		.17	

*Note*: *p < .05, **p < .01, ***p < .001. *R* ^2^ signifies the proportion of variance in foundations explained by the trimmed model. Bootstrapped 95% confidence intervals with 5000 samples.

Abbreviations: MF, Moral Foundations; PO, Political Orientation.

SDO mediated positive indirect paths to all binding foundations, as well as the predicted negative indirect paths to care and fairness. The model based on MFCT foundation scores is an overwhelmingly better fit (see Table 7).

**TABLE 7:** Comparison of model fits as indicated by the Akaike Information Criterion (AIC), Bayesian Information Criterion (BIC) and Log‐Likelihood.

Model	AIC	BIC	LogLik
Trimmed MFQ	11,225.44	11,375.62	−5577.58
Trimmed MFCT	6022.29	6177.03	−2976.25

*Note*: LogLik is for unrestricted models. Model fit is provided for the trimmed models and therefore models differ in the number of parameters (MFQ model has 33 free parameters, MFCT has 34 free parameters). Both AIC and BIC penalize models with more parameters and hence for our purposes this serves as a stricter test of fit for the MFCT model.

### Discussion

In a large diverse general sample, we replicated the MFCT–MFQ association as in study 1. Moreover, we tested the external validity of the MFCT and found it superior to the MFQ. Trade‐offs on the MFCT produce expected associations with established sociopolitical covariates of moral foundations and the MFCT explains more variance than MFQ self‐reports. We take this as strong evidence that the MFCT better reflects the relative strength of activation of foundation representations in response to relevant stimuli.

There are limitations specific to this study. Firstly, we inherit a limitation of the original (Kugler et al., 2014) by employing a single aggregated measure of political orientation. Though we judge this to be appropriate here for the purposes of replication, we encourage future work to include more nuanced measures of political identity. This could include various measures of identity fusion ranging from single‐issue ideology to multi‐issue partisanship or affective polarization, all of which reciprocally interact but can have distinct effects (see Jost et al., 2022 for review; also van Baar & FeldmanHall, 2022). Our design is also correlational and therefore the directions specified in the models both here and in Kugler et al. (2014) (i.e., political orientation as predicting moral foundations), though supported by other work (Hatemi et al., 2019), cannot discern cause and effect. Though prior work has cautioned against overinterpreting direction and fit of mediation models in correlational contexts (Lemmer & Gollwitzer, 2017; Rohrer et al., 2022), the purpose of the mediation model in our work is to test whether established covariate networks are replicated with an alternative measure of moral foundations. To this end, we believe the MFCT outperforms the MFQ and adds to the ongoing discussion about MFT and its explanatory value.

## GENERAL DISCUSSION

We predicted trade‐off patterns between moral foundations would correlate with, but differ from, explicit endorsements of individual foundations. Across four studies, we found that MFQ responses partially mirrored trade‐off patterns in a new task, suggesting that MFQ responses might involve matching the intensity of moral foundation representations *and* aligning with one's political and social identity (cf., Kahneman & Ritov, 1994; Zakharin & Bates, 2021). In contrast, the MFCT, by prompting quick decisions between foundation‐related options, reduces the influence of conscious and self‐presentational biases. Our use of computational modelling to analyse MFCT RTs revealed patterns aligning with MFT predictions; MFCT responses confirmed established sociopolitical correlations, even surpassing MFQ in predictive power and offering insights into the interplay between identity, ideology, personality and moral values.

Critiques of MFT often question the adequacy of MFQ evidence for supporting MFT's foundational structure, with some targeting its five‐factor model (Atari et al., 2022; Jacobsin, 2008; Jost, 2012; Kugler et al., 2014; Schein & Gray, 2015, 2018; Zakharin & Bates, 2021). These debates highlight the need for alternative measurement methods that can converge on moral representations more accurately. Previous research generally measured moral foundations in isolation (Horberg et al., 2009; Inbar et al., 2009; Schnall, Benton, & Harvey, 2008; Schnall, Haidt, et al., 2008; Wheatley & Haidt, 2005; Zhong et al., 2010), through political ideology (Garvey & Ford, 2014; Graham, 2010; Haidt & Graham, 2007; Wright & Baril, 2011), or by manipulating cognitive resources to test effects on explicitly reported foundations (Garvey & Ford, 2014; Napier & Luguri, 2013; Pennycook et al., 2014; Royzman et al., 2014; Van Berkel et al., 2015; Van de Vyver et al., 2016; Wright & Baril, 2011; Yilmaz & Saribay, 2017). We introduce a trade‐off task that isolates and triggers foundational representations, allowing for natural variation in responses and RTs. This not only supports MFT predictions but also expands the measurement scope of moral foundations and more firmly connects MFT to computational models with testable claims about decision‐making processes and underlying representations.

### Limitations

This work has limitations. We adapted items from the Moral Foundations Dictionary (MFD) for our task, which, despite its widespread use and recent updates, has faced criticism for its categorical assignment of words and lack of contextual sensitivity in moral language (Garten et al., 2018; Hopp et al., 2021; Sagi & Dehghani, 2014; Weber et al., 2018). This might introduce noise into task responses, but the ability of MFCT scores to predict identity and attitude‐based correlates better than MFQ suggests resilience to these limitations. Nonetheless, further studies are necessary to validate this. Additionally, advancements in large‐language models present opportunities to refine word‐foundation associations, offering potential for task enhancement.

We measure foundation preferences through rapid judgements, relying on response speed as a proxy for intuition, in line with dual‐process theories (Evans & Stanovich, 2013; Greene et al., 2001; Haidt, 2001; Kahneman, 2011). While speed is a crucial aspect of intuition, there are others (Cushman et al., 2006; Cushman & Greene, 2012; Greene et al., 2004; Haidt, 2001; Moore et al., 2008, 2011). However, sampling models can account for both response caution and evidence accumulation rates, enabling predictions about how experimental factors influence these and how speed‐accuracy trade‐offs vary with task demands (Bogacz et al., 2010; Zhang & Rowe, 2014).

### Future directions

The MFCT serves as a dynamic instrument for examining moral judgements and their underlying influences. Individual preferences for certain moral foundations over others can predict behaviour in situations where multiple foundations are relevant (Dungan et al., 2014; Monroe & Plant, 2019; Waytz et al., 2013). The MFCT offers a direct way to assess these trade‐offs and explore individual differences, to be used alongside other measures (e.g., MFQ for reasoned moral endorsements; moral foundations vignettes (Clifford et al., 2015) for 'concrete' moral scenarios). Moreover, it naturally allows for investigation of manipulations that could shift foundation salience, affect the caution with which participants respond, or both. Future research could also re‐evaluate the longitudinal stability of moral values, which fluctuate more than political identities (Hatemi et al., 2019; Smith et al., 2017). If MFCT‐derived values show greater stability over time, it could challenge the notion that political identity is a more fundamental psychological construct than moral values. Additionally, heritability studies using MFCT might reveal different patterns, given its focus on intuitive processes, potentially reducing environmental influence. All of these would be valuable contributions to ongoing debates over the nature of moral representations and their role in wider cognition.

The MFCT might also shed light on moral inconsistencies within individuals, linking to metacognition and decision‐making research (Colombo et al., 2010; Yeung & Summerfield, 2012). High metacognitive certainty could align with greater information asymmetry in moral foundation sampling, while similar valuation of foundations might reduce metacognitive accuracy despite longer decision times. Our focus on inter‐foundation conflict differs from moral dilemmas involving high‐stakes sacrifices (Crone & Laham, 2015; Cushman & Greene, 2012; Greene et al., 2001–2008; Haidt, 2001; Hopp et al., 2020; Moore et al., 2008, 2011; Piazza et al., 2013). Future enhancements to the MFCT could add high‐stakes contextual framing and explore further forms of moral conflict.

## CONCLUSION

The debate around the nature, number, interrelation and political and personality links of moral values persists. Our novel trade‐off task sheds light on these issues, showing that moral foundations assessed by the MFCT, while moderately correlating with MFQ responses, better explain variations in political identity and associated traits in a theoretically consistent manner. Computational modelling of responses and timings aligns with MFT and broader cognitive sampling models, suggesting distinct moral representations are accessed for judgements. This implies self‐report measures like the MFQ may incorporate ideological and self‐presentational biases, potentially skewing moral psychology discussions and its ties to social cognition.

## AUTHOR CONTRIBUTIONS

**Amrita Ahluwalia‐McMeddes:** Conceptualization; methodology; data curation; formal analysis; investigation; resources; software; writing – review and editing; writing – original draft; visualization. **Adam Moore:** Conceptualization; methodology; software; supervision; formal analysis; visualization; writing – review and editing; writing – original draft. **Calum Marr:** Conceptualization; investigation; writing – original draft. **Zara Kunders:** Investigation.

## CONFLICT OF INTEREST STATEMENT

We have no conflicts of interest to disclose.

## Supporting information


Data S1.

## Data Availability

The data that support the findings of this study are openly available in OSF at http://doi.org/10.17605/OSF.IO/PFR4K.

## Footnotes

^1^
Liberty is proposed as a sixth candidate, centring on reactance towards domination/restriction (Iyer et al., 2012).

2 When equally valued, no drift‐rate differences are expected. Ties have been excluded.

^3^
Several analyses were conducted as part of a doctoral project. Main hypotheses here concern patterns of responses, thus we have included other analyses in the Supporting Information, clearly signposting where these were preregistered. Overall, they do not differ from the same for study 1.

^4^
The UK operates a multiparty system dominated by two parties (Labour and Conservative) that fall into the liberal‐conservative dichotomy. See https://commonslibrary.parliament.uk/research‐briefings/cbp‐7529/.
