# Supplementary material for: Moral trade‐offs reveal foundational representations that predict unique variance in political attitudes
Source: Br J Soc Psychol. 2024 Jul 9;64(1):e12781. doi: 10.1111/bjso.12781 (PMC11588039; doi:10.1111/bjso.12781)
Supplement: Supplementary file 1 — Data S1. [file BJSO-64-0-s001.docx]

# Supplementary Materials

#

# Moral Foundations Conflict Task Items

Table S1 below shows item pairings from the Moral Foundations Conflict Task (MFCT).

To develop MFCT items, an initial list of 80 foundation-relevant words and phrases (16 per foundation) adapted from the Moral Foundations Dictionary (Graham et al., 2009) was drafted to fit into one of four formulations defined by orthogonally crossing valence (virtue/vice) and activity level (active/passive, or alternatively, action/trait) to create four blocks: 1) virtue active – describing active virtue behaviour, and completing the stem ‘It is better to’; (2) virtue passive – passive virtue traits, completing ‘It is better to be’; (3) vice active – active vice behaviour, completing ‘It is worse to’; and (4) vice passive – passive vice traits, completing ‘It is worse to be’.

Each item was scored by three independent coders for valence, i.e. how positive (for foundation virtues) or negative (vices) on a scale of 1 (Not at all positive/negative) to 7 (Very positive/negative), intra-class correlation (*ICC*) = .69, *95% CI*[.58, .77], *F*(79, 160) = 7.53, *p* <.001. Valence ratings from coders were averaged to give each item a valence score.

Item pairings were created to be matched on valence and on length (number of letters). For each inter-foundation combination in each block, item pairings were ordered first to minimise difference in valence score, and then to minimise a difference in length. The top four most closely matched items were chosen to create the final 160 pairings. As a result of this process, five of the original 80 items were dropped because they did not occur in any matched pairs - see Ahluwalia (2015; 2020).

| Table S1. List of item pairs from the MFCT | | | | |  |
| --- | --- | --- | --- | --- | --- |
| *Item 1* | *Item 2* | *Foundation 1* | | *Foundation 2* | |
| *Virtue Active* |  |  |  | |  |
| Comply with people in authority | Protect defenceless animals | Authority | Care | |  |
| Obey elders | Protect defenceless animals | Authority | Care | |  |
| Protect defenceless animals | Respect the traditions of society | Care | Authority | |  |
| Obey the law | Protect defenceless animals | Authority | Care | |  |
| Comply with people in authority | Treat everyone equally | Authority | Fairness | |  |
| Obey elders | Treat everyone equally | Authority | Fairness | |  |
| Be openminded about other people | Comply with people in authority | Fairness | Authority | |  |
| Be openminded about other people | Obey elders | Fairness | Authority | |  |
| Comply with people in authority | Put family before yourself | Authority | Loyalty | |  |
| Obey elders | Put family before yourself | Authority | Loyalty | |  |
| Obey the law | Show love for your country | Authority | Loyalty | |  |
| Put family before yourself | Respect the traditions of society | Loyalty | Authority | |  |
| Act in a modest manner | Comply with people in authority | Purity | Authority | |  |
| Act in a modest manner | Obey elders | Purity | Authority | |  |
| Maintain a clean reputation | Obey the law | Purity | Authority | |  |
| Act in a modest manner | Respect the traditions of society | Purity | Authority | |  |
| Be openminded about other people | Care for vulnerable people | Fairness | Care | |  |
| Care for vulnerable people | Protect others' rights | Care | Fairness | |  |
| Defend vulnerable people | Treat everyone equally | Care | Fairness | |  |
| Care for vulnerable people | Treat everyone fairly | Care | Fairness | |  |
| Protect defenceless animals | Show loyalty to friends | Care | Loyalty | |  |
| Act for the good of the group | Protect defenceless animals | Loyalty | Care | |  |
| Show compassion when others suffer | Show loyalty to friends | Care | Loyalty | |  |
| Act for the good of the group | Show compassion when others suffer | Loyalty | Care | |  |
| Act with integrity | Care for vulnerable people | Purity | Care | |  |
| Act with integrity | Defend vulnerable people | Purity | Care | |  |
| Act with integrity | Show compassion when others suffer | Purity | Care | |  |
| Act in a modest manner | Protect defenceless animals | Purity | Care | |  |
| Show loyalty to friends | Treat everyone equally | Loyalty | Fairness | |  |
| Be openminded about other people | Show loyalty to friends | Fairness | Loyalty | |  |
| Protect others' rights | Show loyalty to friends | Fairness | Loyalty | |  |
| Act for the good of the group | Treat everyone equally | Loyalty | Fairness | |  |
| Act with integrity | Be openminded about other people | Purity | Fairness | |  |
| Act with integrity | Protect others' rights | Purity | Fairness | |  |
| Act with integrity | Treat everyone fairly | Purity | Fairness | |  |
| Act with integrity | Treat everyone equally | Purity | Fairness | |  |
| Act in a modest manner | Put family before yourself | Purity | Loyalty | |  |
| Maintain a clean reputation | Show love for your country | Purity | Loyalty | |  |
| Act for the good of the group | Act in a modest manner | Loyalty | Purity | |  |
| Maintain a clean reputation | Put family before yourself | Purity | Loyalty | |  |
| *Virtue Passive* |  |  |  | |  |
| Respectful | Nurturing | Authority | Care | |  |
| Respectful | Sympathetic | Authority | Care | |  |
| Lawful | Sympathetic | Authority | Care | |  |
| Disciplined | Caring | Authority | Care | |  |
| Respectful | Tolerant | Authority | Fairness | |  |
| Lawful | Tolerant | Authority | Fairness | |  |
| Respectful | Unprejudiced | Authority | Fairness | |  |
| Disciplined | Fair | Authority | Fairness | |  |
| Respectful | Loyal | Authority | Loyalty | |  |
| Obedient | Patriotic | Authority | Loyalty | |  |
| Respectful | Dutiful | Authority | Loyalty | |  |
| Disciplined | Patriotic | Authority | Loyalty | |  |
| Disciplined | Clean | Authority | Purity | |  |
| Respectful | Virtuous | Authority | Purity | |  |
| Lawful | Pure | Authority | Purity | |  |
| Obedient | Pious | Authority | Purity | |  |
| Caring | Tolerant | Care | Fairness | |  |
| Sympathetic | Fair | Care | Fairness | |  |
| Compassionate | Tolerant | Care | Fairness | |  |
| Sympathetic | Unbiased | Care | Fairness | |  |
| Sympathetic | Devoted | Care | Loyalty | |  |
| Caring | Loyal | Care | Loyalty | |  |
| Compassionate | Loyal | Care | Loyalty | |  |
| Compassionate | Dutiful | Care | Loyalty | |  |
| Compassionate | Virtuous | Care | Purity | |  |
| Sympathetic | Clean | Care | Purity | |  |
| Caring | Pure | Care | Purity | |  |
| Compassionate | Pure | Care | Purity | |  |
| Tolerant | Devoted | Fairness | Loyalty | |  |
| Fair | Loyal | Fairness | Loyalty | |  |
| Unbiased | Loyal | Fairness | Loyalty | |  |
| Unbiased | Dutiful | Fairness | Loyalty | |  |
| Unbiased | Virtuous | Fairness | Purity | |  |
| Tolerant | Clean | Fairness | Purity | |  |
| Fair | Pure | Fairness | Purity | |  |
| Unbiased | Pure | Fairness | Purity | |  |
| Patriotic | Pure | Loyalty | Purity | |  |
| Loyal | Clean | Loyalty | Purity | |  |
| Dutiful | Pious | Loyalty | Purity | |  |
| Patriotic | Virtuous | Loyalty | Purity | |  |
| *Vice Active* |  |  |  | |  |
| Do something cruel | Do something illegal | Care | Authority | |  |
| Do something illegal | Harm defenceless animals | Authority | Care | |  |
| Cause chaos or disorder | Do something cruel | Authority | Care | |  |
| Cause chaos or disorder | Harm defenceless animals | Authority | Care | |  |
| Do something illegal | Treat some people differently | Authority | Fairness | |  |
| Cause chaos or disorder | Treat some people differently | Authority | Fairness | |  |
| Act in an obstructive manner | Treat some people differently | Authority | Fairness | |  |
| Cheat to get ahead | Do something illegal | Fairness | Authority | |  |
| Act for selfish reasons | Cause chaos or disorder | Loyalty | Authority | |  |
| Act for selfish reasons | Act in an obstructive manner | Loyalty | Authority | |  |
| Commit treason | Do something illegal | Loyalty | Authority | |  |
| Act for selfish reasons | Do something illegal | Loyalty | Authority | |  |
| Act in an obstructive manner | Behave indecently | Authority | Purity | |  |
| Act in an obscene manner | Do something illegal | Purity | Authority | |  |
| Behave indecently | Show a lack of respect for authority | Purity | Authority | |  |
| Behave indecently | Cause chaos or disorder | Purity | Authority | |  |
| Cheat to get ahead | Make other people suffer | Fairness | Care | |  |
| Cheat to get ahead | Hurt others' feelings | Fairness | Care | |  |
| Do something cruel | Treat some people differently | Care | Fairness | |  |
| Harm defenceless animals | Treat some people differently | Care | Fairness | |  |
| Betray a friend | Do something cruel | Loyalty | Care | |  |
| Betray a friend | Harm defenceless animals | Loyalty | Care | |  |
| Commit treason | Do something cruel | Loyalty | Care | |  |
| Commit treason | Harm defenceless animals | Loyalty | Care | |  |
| Act in an obscene manner | Do something cruel | Purity | Care | |  |
| Act in an obscene manner | Harm defenceless animals | Purity | Care | |  |
| Act in an obscene manner | Hurt others' feelings | Purity | Care | |  |
| Behave indecently | Do something cruel | Purity | Care | |  |
| Commit treason | Treat some people differently | Loyalty | Fairness | |  |
| Betray a friend | Treat some people differently | Loyalty | Fairness | |  |
| Betray a friend | Cheat to get ahead | Loyalty | Fairness | |  |
| Act for selfish reasons | Treat some people differently | Loyalty | Fairness | |  |
| Act in an obscene manner | Treat some people differently | Purity | Fairness | |  |
| Behave indecently | Treat some people differently | Purity | Fairness | |  |
| Act in an obscene manner | Cheat to get ahead | Purity | Fairness | |  |
| Behave indecently | Cheat to get ahead | Purity | Fairness | |  |
| Act in an ungodly way | Insult your country | Purity | Loyalty | |  |
| Act for selfish reasons | Behave indecently | Loyalty | Purity | |  |
| Act in an obscene manner | Commit treason | Purity | Loyal | |  |
| Act for selfish reasons | Act in an obscene manner | Loyalty | Purity | |  |
| *Vice Passive* |  |  |  | |  |
| Undisciplined | Cruel | Authority | Care | |  |
| Undisciplined | Neglectful | Authority | Care | |  |
| Subversive | Cruel | Authority | Care | |  |
| Disobedient | Harmful | Authority | Care | |  |
| Undisciplined | Biased | Authority | Fairness | |  |
| Undisciplined | Intolerant | Authority | Fairness | |  |
| Subversive | Biased | Authority | Fairness | |  |
| Rebellious | Unfair | Authority | Fairness | |  |
| Undisciplined | Unpatriotic | Authority | Loyalty | |  |
| Undisciplined | Unfaithful | Authority | Loyalty | |  |
| Disobedient | Disloyal | Authority | Loyalty | |  |
| Subversive | Unfaithful | Authority | Loyalty | |  |
| Rebellious | Dirty | Authority | Purity | |  |
| Disobedient | Promiscuous | Authority | Purity | |  |
| Undisciplined | Sinful | Authority | Purity | |  |
| Rebellious | Promiscuous | Authority | Purity | |  |
| Cruel | Unfair | Care | Fairness | |  |
| Aggressive | Biased | Care | Fairness | |  |
| Harmful | Biased | Care | Fairness | |  |
| Harmful | Intolerant | Care | Fairness | |  |
| Cruel | Disloyal | Care | Loyalty | |  |
| Neglectful | Disloyal | Care | Loyalty | |  |
| Cruel | Selfish | Care | Loyalty | |  |
| Aggressive | Unfaithful | Care | Loyalty | |  |
| Cruel | Promiscuous | Care | Purity | |  |
| Neglectful | Promiscuous | Care | Purity | |  |
| Aggressive | Sinful | Care | Purity | |  |
| Harmful | Sinful | Care | Purity | |  |
| Biased | Disloyal | Fairness | Loyalty | |  |
| Intolerant | Disloyal | Fairness | Loyalty | |  |
| Biased | Selfish | Fairness | Loyalty | |  |
| Unfair | Unfaithful | Fairness | Loyalty | |  |
| Biased | Promiscuous | Fairness | Purity | |  |
| Intolerant | Promiscuous | Fairness | Purity | |  |
| Unfair | Sinful | Fairness | Purity | |  |
| Unjust | Sinful | Fairness | Purity | |  |
| Unfaithful | Promiscuous | Loyalty | Purity | |  |
| Disloyal | Sinful | Loyalty | Purity | |  |
| Selfish | Sinful | Loyalty | Purity | |  |
| Unpatriotic | Promiscuous | Loyalty | Purity | |  |

# Study 1

##

## RT Cut-offs

Criteria to identify RT error trials on the MFCT is outlined in Ahluwalia (2020). To summarise this approach, we reviewed RT cut-offs employed in previous research, applying the upper bound of 15 seconds used by Graham (2010) in an analogous trade-off task. However, given the relative complexity of the stimuli in the MFCT relative to this previous trade-off task (which employed a lower bound of >150ms) we opted for 400ms.

## Drift Diffusion Model

As indicated in the main text, the total possible parameter space for all configurations of the model is too large and beyond the scope of our work here. We tested multiple versions, primarily focused on the utility of valence, trait/action, and MFCT ranks as predictors of drift rate. In Table S2, we report the model configurations and deviance information criteria (DIC) for each of those we tested. Ultimately, we chose the more parsimonious model presented in the main text. Model 3, below, does show a superior DIC, but when comparing posterior predictive accuracy there was not a meaningful difference across quantiles, the inference related to the MFCT ranks was unchanged compared to the final model, and given that it was significantly more complex, we elected to report the simpler one.

Table S2. Various configurations of the drift diffusion model applied to response time data from the MFCT task in study 1

| Model Name | Configuration | DIC Value |
| --- | --- | --- |
| Final Model | *v* ~ 1 + Valence + MFCTRank  *a* ~ 1 + Valence * Action  *t* depends on Action | 42295.31 |
| Model 2 | *v* ~ 1 + Valence * MFCTRank  *a* ~ 1 + Valence * Action  *t* depends on Action | 42300.74 |
| Model 3 | *v* ~ 1 + Valence * Action * MFCTRank  *a* ~ 1 + Valence * Action  *t* depends on Action | 42287.26 |

*Note.* *v* = drift rate, *a* = caution/response threshold separation, *t* = non-decision/reading time.

##

## Internal reliability and stability across blocks

Within-participant split-half correlations were calculated for MFCT scores across the task and across blocks (see Table S3). Reliability for the full task, and across blocks, is acceptable, but lower for the active blocks, likely reflecting greater item complexity.

All analyses of how block structure on the MFCT affects responses and RTs is available in Ahluwalia (2020). To summarise these results, there was no evidence that responses (MFCT scores) differed across valence or action blocks (or their interaction). An interaction between valence and action did affect RT – it was quicker to make choices between passive items in the virtue block (thus subsequent RT analyses included random intercepts for valence and action where applicable).

| Table S3. Bootstrapped split-half reliability across blocks for study 1 | | | | | |
| --- | --- | --- | --- | --- | --- |
|  | *r_Boot_* | Bias | 95% CI of *r* | *SE r_Boot_* |  |
| *N =* 75 | | | | | |
| Full Task | .85 | .002 | [.81, .89] | .02 |  |
| Vice | .83 | .01 | [.77, .87] | .02 |  |
| Virtue | .79 | -.02 | [.73, .89] | .04 |  |
| Active | .69 | .05 | [.58, .70] | .03 |  |
| Passive | .80 | -.002 | [.74, .87] | .03 |  |
| *Note.* Within-participant split-half correlations were calculated and adjusted using the Spearman-Brown formula to get an estimate of reliability of the full MFCT (Eisinga et al., 2013; Kaplan & Saccuzzo, 2001). This process was then bootstrapped with 5,000 iterations. | | | | | |

## Exploratory RT analyses

Several exploratory analyses of RT are reported in Ahluwalia (2020). These include multilevel models of mean RT and Ex-Gaussian decomposition of the RT distribution (fitting *μ* – the mean; *σ* – standard deviation of the Gaussian distribution; and *τ* – the exponential parameter producing the skewed tail). All RTs in the models reported below refer to response times in completing the MFCT - where models are labelled ‘MFQ’, this refers to how rank ordering has been defined for these models, i.e. where the ranked order of preferences is based on MFQ responses.

Following theoretical precedents for interpretation (Heathcote et al., 1991; Lacouture & Cousineau, 2008; Luce, 1986; McGill, 1963; Moore et al., 2011), the *μ* parameter reflects the transduction component, i.e. time required by sensory processes and to physically make responses, while the *τ* parameter represents the decision component, i.e. time required to decide which response to make. Thus, increased conflict should produce a greater *τ* estimate.

We anticipated that both mean RT and *τ* would increase for foundations closer in value (implemented as distance in the rank order of preferences for foundations as indicated by the MFQ and on the MFCT). Contrary to DDM results reported in main text, the rank preferences here represent the relative – rather than the overall – value of foundations in a choice, i.e., choices between 1^st^ and 2^nd^ ranked foundations and 4^th^ and 5^th^ ranked foundations would both be categorised as 1 Rank Apart (RA). This lack of resolution is one reason why we report DDM results in the main text.

Though not significant for all comparisons, we generally found that 1 RA choices corresponded with higher mean RT, with a decreasing pattern evident for ranks based on the MFCT. *τ* also tended to decrease for foundations further apart in value on the MFCT, consistent with expectations that this corresponds with making easier, less conflicting decisions. Generally, these effects were small (see Table S4, Figure S1 and Figure S2).

| Table S4*.* Predicting RT, *μ,* and *τ* from ranks apart on the MFQ and MFCT for study 1 | | | | | | |
| --- | --- | --- | --- | --- | --- | --- |
|  | *Models* | | | | | |
|  | MFQ | | | MFCT | | |
|  | log RT | log *μ* | log *τ* | log RT | log *μ* | log *τ* |
|  | | | | | | |
| *Fixed effects* |  |  |  |  |  |  |
| Intercept | .02 (.11) | .003 (.09) | .01 (.10) | -.03 (.11) | -.03 (.09) | -.001 (.10) |
| Ranks Apart |  |  |  |  |  |  |
| 1 RA v. 2, 3, 4 | .10^**^ (.04) | .11 (.10) | .07 (.09) | .21^***^ (.04) | .15 (.10) | .25^**^ (.08) |
| 2 RA v. 3, 4 | .04 (.04) | -.16 (.11) | .26^**^ (.10) | .15^***^ (.04) | .09 (.11) | .16^†^ (.09) |
| 3 RA v. 4 | -.05 (.05) | -.02 (.14) | -.09 (.12) | .14^*^ (.05) | .30^*^ (.13) | -.13 (.11) |
|  | | | | | | |
| Marg. *R^2^* / Cond. *R^2^* | .003 / .93 | .01 / .52 | .01 / .61 | .01 / .92 | .01 / .49 | .02 / .64 |
| LogLik | -159 | -335 | -317 | -187 | -360 | -327 |
| AIC | 330 | 683 | 646 | 386 | 731 | 665 |
| BIC | 352 | 704 | 668 | 408 | 753 | 687 |
|  | | | | | | |
| *Note.* ^†^ *p* < .10, * *p* < .05, ** *p* < .01, *** *p* < .001. Number of observations = 265 (MFQ) and 280 (MFCT). RA – Ranks Apart. Fixed effects for separate models predicting log RT, *μ* and *τ* for ranks apart based on the MFQ and the MFCT*.* Outcome variables have been standardised. Helmert coding compares each rank apart category to the mean of the subsequent categories. Due to infrequent occurrence, models were fit with 0 RA category dropped, meaning that 32 participants were excluded from MFQ models, and 19 had from MFCT models. *SE* is provided in parentheses. Ex-Gaussian parameters were estimated (5,000 iterations) using the maximum likelihood method with *timefit* in the *retimes* package (version 0.1-2) (Massidda, 2013). | | | | | | |

Figure S1. Ranks apart on MFQ predicting RT (a), μ (b) and τ (c) for study 1. Error bars denote 95% CIs, corrected for within-subject designs based on Morey (2008).

Figure S2. Ranks apart on MFCT predicting RT (a), μ (b) and τ (c) for study 1. Error bars denote 95% CIs, corrected for within-subject designs based on Morey (2008).

# Study 2a

## RT Exclusions

Applying the same inclusion criteria as in study 1, a total of twenty-five trials on the MFCT (<.01%) were removed across 16 subjects, leaving a total of 15,829 trials (*M_RT Control_* = 2280ms; *SD_RT Control_ =* 1467ms; *M_RT Load_* = 2331ms; *SD_RT Load_ =* 1502ms). For RT analyses with this and study 2b data like those presented above for study 1, see Ahluwalia (2020).

## Correlations

Table S5 shows mean responses and correlation across foundations. Pearson’s *r* correlations were large (or approaching) for all foundations except fairness.

| Table S5. Descriptive statistics and Pearson correlations for study 2a variables | | | | | | | | | | | |
| --- | --- | --- | --- | --- | --- | --- | --- | --- | --- | --- | --- |
|  | *M* | *SD* | 1 | 2 | 3 | 4 | 5 | 6 | 7 | 8 | 9 |
| 1. Care-MFQ | 3.9 | 0.6 | - |  |  |  |  |  |  |  |  |
| 2. Fairness-MFQ | 3.9 | 0.5 | .33** | - |  |  |  |  |  |  |  |
| 3. Authority-MFQ | 2.2 | 0.7 | -.01 | .10 | - |  |  |  |  |  |  |
| 4. Loyalty-MFQ | 2.4 | 0.8 | .12 | -.04 | .62*** | - |  |  |  |  |  |
| 5. Purity-MFQ | 1.7 | 1.0 | .15 | .04 | .62*** | .50*** | - |  |  |  |  |
| 6. Care-MFCT | .72 | .14 | **.46***** | .08 | -.51*** | -.40*** | -.33** | - |  |  |  |
| 7. Fairness-MFCT | .58 | .14 | -.19 | **.24** | -.36** | -.40*** | -.46*** | .02 | - |  |  |
| 8. Authority-MFCT | .39 | .10 | -.26† | -.12 | **.54***** | .22 | .30* | -.54*** | -.37** | - |  |
| 9. Loyalty-MFCT | .44 | .10 | .02 | -.19 | .19 | **.50***** | .10 | -.22 | -.50*** | .05 | - |
| 10. Purity-MFCT | .37 | .13 | -.11 | -.12 | .38*** | .32** | **.58***** | -.53*** | -.44*** | .17 | -.04 |
| *Note.* † *p* < .10, * *p* < .05, ** *p* < .01, *** *p* < .001. MFQ scores are on a 0 to 5 scale, MFCT scores are on a 0 to 1 scale. *p*-values corrected for multiple comparisons (Bonferroni). Correlations between the same foundations on the MFQ and on the MFCT have been highlighted in bold. | | | | | | | | | | | |

## Self-reported tone-counting performance

Load participants completed four items to gauge the perceived performance and difficulty on the tone-counting task: ‘How difficult did you find the decision making task?’; ‘How much did having to count the tones interfere with your ability to choose between the words or phrases?’; ‘How accurate do you think you were in the counting tones?’; and ‘How much do you think your responses on the decision making task reflected your beliefs/values?’(scaled from 1 to 10).

Participants reported middling accuracy on counting the tones, *M* = 5.7, *SD* = 1.9, and tended to feel that this had interfered with their ability to make choices on the MFCT, *M* = 7.7, *SD* = 1.2. Participants also found the joint task difficult, *M* = 6.8, *SD* = 1.7, but felt responses were reflective of their beliefs and values, *M* = 7.1, *SD* = 1.50. However, these perceptions about performance did not correlate with actual performance in the number of errors made, -.27 < *r*s < .12, *p*s > .10.

#

# Study 2b

##

## RT Exclusions

## Again applying the same criteria as previous, a total of sixty-one trials (<.01%) were removed across 9 subjects. One subject was removed because more than 10% of their trials were eliminated, leaving a total of 14,224 trials (*M_RT Control_* = 2187ms; *SD_RT Control_ =* 1238ms; *M_RT Alcohol_* = 2421ms; *SD_RT Alcohol_ =* 1364ms). The final sample comprised 89 participants (64% females, *M_age_* = 23.76; *SD =* 4.53), with 43 in the control, and 46 in the alcohol condition.

**Alcohol dose**

Alcohol doses comprised 1 part vodka to 3 parts mixer (lemonade), calculated for each participant based on body-weight (in kilograms) and sex (0.20g/kg for female and 0.25g/kg for male participants) to achieve 0.03 BAC. The vodka used contained 7.41g of alcohol in every 25ml (1 unit). Participants consumed an average of 42.78ml (females) and 66.41ml (males) of vodka.

## Correlations

Table S6 shows mean responses and correlation across foundations. Pearson’s *r* correlations for all foundations were either large (care, authority, purity) or medium (fairness and loyalty).

| Table S6. Descriptive statistics and Pearson correlations for study 2b variables | | | | | | | | | | | |
| --- | --- | --- | --- | --- | --- | --- | --- | --- | --- | --- | --- |
|  | *M* | *SD* | 1 | 2 | 3 | 4 | 5 | 6 | 7 | 8 | 9 |
| 1. Care-MFQ | 3.7 | 0.8 | - |  |  |  |  |  |  |  |  |
| 2. Fairness-MFQ | 3.8 | 0.7 | .51*** | - |  |  |  |  |  |  |  |
| 3. Authority-MFQ | 2.3 | 0.8 | -.03 | -.22 | - |  |  |  |  |  |  |
| 4. Loyalty-MFQ | 2.4 | 0.8 | -.04 | -.13 | .65*** | - |  |  |  |  |  |
| 5. Purity-MFQ | 1.7 | 1.0 | .16 | -.18 | .63*** | .66*** | - |  |  |  |  |
| 6. Care-MFCT | .68 | .15 | **.58***** | .26 | -.30* | -.33* | -.23 | - |  |  |  |
| 7. Fairness-MFCT | .62 | .15 | -.07 | **.38**** | -.48*** | -.47*** | -.59*** | .16 | - |  |  |
| 8. Authority-MFCT | .40 | .12 | -.33* | -.21 | **.52***** | .23 | .17 | -.41*** | -.31* | - |  |
| 9. Loyalty-MFCT | .45 | .12 | -.26 | -.21 | .08 | **.36**** | .11 | -.48*** | -.43*** | .00 | - |
| 10. Purity-MFCT | .35 | .15 | -.03 | -.29* | .29† | .32* | **.59***** | -.42*** | -.55*** | -.10 | .08 |
| *Note.* † *p* < .10, * *p* < .05, ** *p* < .01, *** *p* < .001. MFQ scores are on a 0 to 5 scale, MFCT scores are on a 0 to 1 scale. *p*-values corrected for multiple comparisons (Bonferroni). Correlations between the same foundations on the MFQ and on the MFCT have been highlighted in bold. | | | | | | | | | | | |

## Alcohol consumption

Participants in the alcohol condition were asked how often they consumed alcohol (Once a month or less / 2 to 4 times a month / 2 to 3 times a week / Four or more times a week), and how much they consumed per week (One to 14 units / 14 to 20 units / More than 20 units). 23.9% of participants indicated 2 to 3 times per week, 50.0% reported 2 to 4 times per month, and 19.6% once a month or less. One participant reported drinking more than four times per week, and was removed from subsequent analyses of alcohol consumption, but not from main analyses for this study. Most participants reported consuming less than 14 units per week (80.4%), with the remaining participants consuming less than 20 units.

Correlations between the MFQ and MFCT differed based on how often participants drank, Kruskal Wallis *H*(2) = 6.52, *p* < .05. Pairwise comparisons suggested this difference to be driven by the most infrequent drinkers having lower MFQ-MFCT consistency, with lower correlations (*p* = .05) when participants reported once a month or less (*M* = .43, *SD* = .32) compared to participants that reported 2 to 4 times per month (*M* = .73, *SD* = .22). There were no other significant pairwise comparisons (*p*s > .19). There were no evident differences in correlations based on how many units were consumed per week, Wilcoxon *W* = 116.00, *p* > .05.

# Study 3

##

## Internal reliability and stability across blocks

Table S7 shows split-half reliability coefficients, showing similar reliability as in study 1.

To summarise results from block analyses (Ahluwalia, 2020), as in study 1, there was no evidence that responses differed across valence or action blocks (or their interaction), though RT was quicker for choices between passive items in the virtue block.

| Table S7. Bootstrapped split-half reliability across blocks for study 3 | | | | | |
| --- | --- | --- | --- | --- | --- |
|  | *r_Boot_* | Bias | 95% CI of *r* | *SE r_Boot_* |  |
| *N =* 700 | | | | | |
| Full Task | .79 | .03 | [.73, .80] | .02 |  |
| Vice | .77 | .02 | [.71, .79] | .02 |  |
| Virtue | .75 | -.02 | [.70, .83] | .03 |  |
| Active | .65 | .09 | [.50, .62] | .03 |  |
| Passive | .79 | .004 | [.75, .83] | .02 |  |
| *Note.* Following the same procedure as for study 1. Bootstrapped with 5,000 iterations. | | | | | |

## Predicting RT on the MFCT

###

### Preregistered analysis

All preregistered results are also reported in Ahluwalia (2020). We predicted that differences in foundation endorsement would predict greater conflict in decisions between closely valued foundations, as measured by mean RT scores and fitted *τ* parameters, and anticipated that these effects would be small for models based on MFQ scores, and small to moderate for models based on MFCT scores.

We preregistered several analyses that differentially operationalised the relative value of foundations. Firstly, we preregistered multilevel models (see Table S8) predicting RT from both a linear (*x*) and quadratic (*x^2^*) term of difference in standardised MFQ and MFCT scores. For both, RT increased the lower the difference in value, though effects were small.

Secondly, we preregistered two sets of multilevel models (see Table S9) predicting RT, *τ*, and *μ* based on distance in ranked preferences for foundations (as indicated by the MFQ and MFCT) – the equivalent of similar (exploratory) analyses reported for study 1. Decreasing trends in RT and τ, as the number of ranks apart increased, were more apparent than for study 1 and were evident in mean RT in all comparisons, and for nearly all in *τ*. The exception was 0 RA (equally valued) choices resulted in lower *τ* (significant for MFQ model, anecdotally for MFCT model) relative to further apart choices, suggesting lower conflict. Patterns were less consistent in *μ*, though still suggested a decreasing trend (see Figure S3 and Figure S4).

Generally, these findings are consistent with those in study 1, with cleaner effects, reported above, indicating that foundation preference is reflected in RTs on the MFCT, and that this appears to track with greater conflict in decisions between more closely valued foundations.

| Table S8. Predicting RT from difference in MFQ and MFCT scores for study 3 | | |
| --- | --- | --- |
|  | *Models* | |
|  | log RT | |
|  | | |
| *Fixed effects* |  |  |
| Intercept | .06^*^ (.02) | .11^***^ (.03) |
| Difference in MFQ Scores | -.04^***^ (.01) |  |
| Difference in MFQ Scores^2^ | -.01^**^ (.003) |  |
| Difference in MFCT Scores |  | -.04^***^ (.01) |
| Difference in MFCT Scores^2^ |  | -.02^***^ (.003) |
|  | | |
| *Random effects* |  |  |
| By Subject - *σ* |  |  |
| Intercept | .63 | .64 |
| Foundation Combination | < .001 | < .001 |
| Valence | < .001 | < .001 |
| Action | .34 | .33 |
| Residual | .70 | .70 |
| Marginal *R^2^* / Conditional *R^2^* | .004 / .52 | .01 / .52 |
| LogLik | -129,617 | -129,152 |
| AIC | 259,251 | 258,319 |
| BIC | 259,328 | 258,396 |
|  | | |
| *Note.* ^†^ *p* < .10, * *p* < .05, ** *p* < .01, *** *p* < .001. Number of observations = 111,709. Fixed and random effects for separate models predicting log RT. Outcome variables have been standardised. Quadratic models fit terms for difference in scores (x) and squared difference in scores (x^2^) between foundations in a trial. To preserve a minimum value of 0 interpretable as no difference between scores for the quadratic term, difference predictors in these models were scaled by SD without centring. For fixed effects, *SE* is provided in parentheses. | | |

| Table S9*.* Predicting RT, *μ,* and *τ* from ranks apart on the MFQ and MFCT for study 3 | | | | | | |
| --- | --- | --- | --- | --- | --- | --- |
|  | *Models* | | | | | |
|  | MFQ | | | MFCT | | |
|  | log RT | log *μ* | log *τ* | log RT | log *μ* | log *τ* |
|  | | | | | | |
| *Fixed effects* |  |  |  |  |  |  |
| Intercept | -.02 (.04) | .004 (.03) | -.03 (.03) | .03 (.04) | .04 (.03) | -.02 (.03) |
| Ranks Apart |  |  |  |  |  |  |
| 0 RA v. 1, 2, 3, 4 | .13^***^ (.02) | .20^***^ (.03) | -.13^**^ (.04) | .25^***^ (.02) | .27^***^ (.05) | -.01 (.06) |
| 1 RA v. 2, 3, 4 | .14^***^ (.01) | .07^**^ (.02) | .19^***^ (.03) | .21^***^ (.01) | .15^***^ (.02) | .20^***^ (.03) |
| 2 RA v. 3, 4 | .13^***^ (.01) | .03 (.03) | .20^***^ (.03) | .20^***^ (.01) | .09^***^ (.02) | .23^***^ (.03) |
| 3 RA v. 4 | .10^***^ (.02) | .03 (.03) | .09^*^ (.04) | .16^***^ (.01) | .04 (.03) | .25^***^ (.04) |
|  | | | | | | |
| *Random effects* |  |  |  |  |  |  |
| By Subject - *σ* |  |  |  |  |  |  |
| Intercept | .97 | .87 | .75 | .96 | .87 | .76 |
| Residual | .26 | .51 | .65 | .25 | .48 | .64 |
| Marginal *R^2^* / Conditional *R^2^* | .01 / .94 | .01 / .74 | .01 / .57 | .02 / .94 | .01 / .77 | .02 / .60 |
| LogLik | -1,560 | -2,914 | -3,353 | -1,539 | -2,842 | -3,366 |
| AIC | 3,135 | 5,843 | 6,719 | 3,091 | 5,698 | 6,746 |
| BIC | 3,176 | 5,884 | 6,761 | 3,133 | 5,739 | 6,788 |
|  | | | | | | |
| *Note.* ^†^ *p* < .10, * *p* < .05, ** *p* < .01, *** *p* < .001. Number of observations = 2,738 (MFQ) and 2,793 (MFCT). RA – Ranks Apart. Fixed and random effects for separate models predicting log RT, *μ* and *τ* for ranks apart based on the MFQ and the MFCT*.* Outcome variables have been standardised. Helmert coding compares each rank apart category to the mean of the subsequent categories. In contrast to study 1, 0 RA category was included. *SE* is provided in parentheses. Ex-Gaussian parameters were estimated (5,000 iterations) using the maximum likelihood method with *timefit* in the *retimes* package (version 0.1-2) (Massidda, 2013). | | | | | | |

Figure S3. Ranks apart on MFQ predicting RT (a), μ (b), and τ (c) for study 3. Error bars denote 95% CIs, corrected for within-subject designs based on Morey (2008).

Figure S4. Ranks apart on MFCT predicting RT (a), μ (b), and τ (c) for study 3. Error bars denote 95% CIs, corrected for within-subject designs based on Morey (2008).

## Mediation Models

### Preregistered analysis

##### Saturated models

In addition to preregistered mediation analyses reported in the main text, we present here the saturated models for the MFQ (illustrated in Figure S5 below) and MFCT (Figure S6 below) for trimmed models in the main text.

Figure S5. Saturated path model showing relationships between political orientation, SDO, RWA, and foundations on the MFQ. Path coefficients are standardised regression coefficients of the full model. Broken lines indicate non-significant paths at *p* > .05.

Figure S6. Saturated path model showing relationships between political orientation, SDO, RWA, and foundations on the MFCT. Path coefficients are standardised regression coefficients of the full model. Broken lines indicate non-significant paths at *p* > .05.

### Exploratory analysis

We fit two sets of exploratory mediation models that further support the interpretation in the main text that the MFCT accounts for unique, non-overlapping variance in foundation preferences relative to the MFQ. We regressed MFQ scores on MFCT scores, and vice versa, storing the residuals as new variables. The residuals thus represent unique variance in MFQ scores not explained by the MFCT, as well as unique variance in MFCT scores not explained by the MFQ. Using these new sets of variables, labelled with ‘Unique’ in the illustrations below, we built two further sets of models.

#### MFQ Unique Model

Saturated model and trimmed models are illustrated in Figure S7 and Figure S8. Table S10 shows direct and indirect paths from political orientation to unique variance in foundations on the MFQ. RWA mediated paths from political orientation to all five foundations, whilst SDO mediated paths to care, fairness, and purity. Relative to their counterparts in the main MFQ model (Figure S5), paths from RWA to care, fairness, loyalty, authority, and purity remained significant, with paths to care and fairness increasing in strength, suggesting that the unique variance in MFQ scores for these foundations is more strongly positively associated with RWA. Associations between SDO and care and fairness also remained significant. However, authority was no longer significantly predicted by SDO suggesting that the variance in endorsement of authority that had been explained in the main MFQ model is also captured by the MFCT. Whereas purity had not been significant in the main MFQ model, it was significant in this model in the opposite direction. Associations between political orientation and loyalty/authority remained significant and became marginally significant for care.

Figure S7. Saturated path model showing relationships between political orientation, SDO, RWA, and unique variance in foundations on the MFQ. Path coefficients are standardised regression coefficients of the full model. Broken lines indicate non-significant paths at *p* > .05.

Figure S8. Trimmed path model showing relationships between political orientation, SDO, RWA, and unique variance in foundations on the MFQ. Path coefficients are standardised regression coefficients of the full model. Broken lines indicate non-significant paths at *p* > .05.

| Table S10. Direct and indirect paths from political orientation to foundations on unique variance in the MFQ (study 3) | | | | | | | | | | |  |
| --- | --- | --- | --- | --- | --- | --- | --- | --- | --- | --- | --- |
|  | Care Unique | | Fairness Unique | | Loyalty Unique | | Authority Unique | | Purity Unique | | |
|  | *β* | 95% CI | *β* | 95% CI | *β* | 95% CI | *β* | 95% CI | *β* | 95% CI | |
| *Direct paths* | | | | | | | | | | | |
| PO → MF | -.08^†^ | [-.16, .01] | .00 | [-.08, .09] | .10^*^ | [.02, .18] | .13^***^ | [.07, .20] | .03 | [-.04, .10] | |
| RWA → MF | .22^***^ | [.13, .30] | .22^***^ | [.13, .30] | .39^***^ | [.30, .48] | .28^***^ | [.22, .35] | .58^***^ | [.51, .64] | |
| SDO → MF | - .35^***^ | [-.43, -.27] | -.47^***^ | [-.54, -.39] | – | – | – | – | -.10^**^ | [-.16, -.04] | |
| *Indirect paths* | | | | | | | | | | | |
| PO → RWA → MF | .11^***^ | [.07, .15] | .11^***^ | [.06, .15] | .19^***^ | [.15, .24] | .14^***^ | [.11, .17] | .29^***^ | [.24, .34] | |
| PO → SDO → MF | -.19^***^ | [-.23, -.14] | -.24^***^ | [-.30, -.19] | – | – | – | – | -.05^**^ | [-.08, -.02] | |
| *R^2^* | .14 |  | .20 |  | .22 |  | .20 |  | .36 |  | |
| *Note.* ^†^ *p* < .10, * *p* < .05, ** *p* < .01, *** *p* < .001. PO – Political Orientation, MF – Moral Foundations. *R^2^* signifies the proportion of variance in foundations explained by the trimmed model. Bootstrapped 95% confidence intervals with 5,000 resamples. | | | | | | | | | | |  |

#### MFCT Unique Model

Saturated model and trimmed models are illustrated in Figure S9 and Figure S10. Table S11 shows direct and indirect paths from political orientation to unique variance in foundations on the MFCT. RWA mediated paths from political orientation to care, fairness, and authority, whilst SDO mediated paths to loyalty, authority, and purity. Compared to the main MFCT model (Figure S6), SDO remained positively associated with authority, loyalty, and purity, suggesting that the MFCT captures unique variance in these foundations predicted by SDO. Paths from RWA to care, fairness, and authority remained significant, suggesting that RWA predicts unique variance in these MFCT foundation scores. Whereas loyalty had not been significant in the main MFCT model, it was marginally significant in this model in the opposite direction. There were no significant associations between SDO and care/fairness, or purity and RWA, suggesting that the variance in these predicted by SDO/RWA is also captured in the MFQ. The association between political orientation and fairness remained significant, but this was not the case for loyalty/authority.

Comparing models, we see that fit is similar in both, with slightly better fit for the unique MFQ model (see Table S12). Despite this, the unique MFCT model reflects patterns in the nomothetic network connecting these variables that are theoretically coherent with MFT, while the unique MFQ model does not. Furthermore, model fit for the unique MFQ model does not change substantially from the full MFQ model (see main text, Table 7), whereas excluding the variance shared with the MFQ worsens fit for the MFCT. Method variance may provide a partial explanation for this drop in the variance for the unique MFCT model – the MFQ has a measurement advantage, being of similar format to the self-report scales used for political orientation, RWA and SDO. Regardless, and even after accounting for variance shared with the MFQ, the MFCT predicts unique and theoretically coherent structured relationships.

Figure S9. Saturated path model showing relationships between political orientation, SDO, RWA, and unique variance in foundations on the MFCT. Path coefficients are standardised regression coefficients of the full model. Broken lines indicate non-significant paths at *p* > .05.

Figure S10. Trimmed path model showing relationships between political orientation, SDO, RWA, and unique variance in foundations on the MFCT. Path coefficients are standardised regression coefficients of the full model. Broken lines indicate non-significant paths at *p* > .05.

| Table S11. Direct and indirect paths from political orientation to foundations on unique variance in the MFCT (study 3) | | | | | | | | | | |
| --- | --- | --- | --- | --- | --- | --- | --- | --- | --- | --- |
|  | Care Unique | | Fairness Unique | | Loyalty Unique | | Authority Unique | | Purity Unique | |
|  | *β* | 95% CI | *β* | 95% CI | *β* | 95% CI | *β* | 95% CI | *β* | 95% CI |
| *Direct paths* | | | | | | | | | | |
| PO → MF | .02 | [-.06, .09] | -.16^***^ | [-.23, -.10] | .01 | [-.07, .09] | .00 | [-.07, .08] | .03 | [-.05, .11] |
| RWA → MF | -.34^***^ | [-.41, -.27] | -.41^***^ | [-.47, -.35] | – | – | .10^**^ | [.03, .17] | – | – |
| SDO → MF | – | – | – | – | .17^***^ | [.09, .25] | .08^*^ | [.01, .15] | -.17^***^ | [.10, -.25] |
| *Indirect paths* | | | | | | | | | | |
| PO → RWA → MF | -.17^***^ | [-.21, -.13] | -.20^***^ | [-.24, -.17] | – | – | .05^**^ | [.02, .09] | – | – |
| PO → SDO → MF | – | – | – | – | .09^***^ | [.05, .14] | .04^*^ | [.00, .08] | .09^***^ | [.05, .14] |
| *R^2^* | .13 |  | .30 |  | .04 |  | .04 |  | .04 |  |
| *Note.* ^†^ *p* < .10, * *p* < .05, ** *p* < .01, *** *p* < .001. PO – Political Orientation, MF – Moral Foundations. *R^2^* signifies the proportion of variance in foundations explained by the trimmed model. Bootstrapped 95% confidence intervals with 5,000 resamples. | | | | | | | | | | |

| Table S12. Comparison of model fits as indicated by the Akaike Information Criterion (AIC), Bayesian Information Criterion (BIC) and Log-Likelihood | | | |
| --- | --- | --- | --- |
| Model | AIC | BIC | LogLik |
| Trimmed MFQ Unique | 11192.48 | 11342.67 | -5562.97 |
| Trimmed MFCT Unique | 11999.48 | 12140.56 | -5965.68 |
| *Note.* LogLik is for unrestricted model, MFQ trimmed model has 33 free parameters, MFCT trimmed model has 31 free parameters | | | |

#### Political orientation

##### Preregistered analysis

We preregistered two hypotheses specific to effects of political orientation. We expected that higher conservatism, and thus endorsement of a wider array of foundations, would predict lower correlations between MFQ and MFCT scores, indicating lower consistency in foundation endorsement; and higher mean RT and *τ* values in the MFCT, indicating greater levels of conflict across trials. We found that the former was supported, correlations between MFQ and MFCT scores decreased with higher conservatism. However, more conservative participants tended to have marginally lower mean RT and significantly lower mean *τ*, suggesting that they experience less conflict in decisions between foundations on the MFCT. These results are presented in Table S13 below and further discussed in Ahluwalia (2020).

| Table S13. Predicting correlation coefficients between MFQ and MFCT, and RT and *τ* on the MFCT, from political orientation for study 3 | | | |
| --- | --- | --- | --- |
|  | *Models* | | |
|  | Correlation coefficient (*r_τ_*) | log RT | log *τ* |
|  | | | |
| Intercept | -.00 (.04) | -.00 (.04) | -.00 (.04) |
| Political Orientation | -.25^***^ (.04) | -.06^†^ (.04) | -.11^**^ (.04) |
|  | | | |
| *R^2^* | .06 | .004 | .01 |
| Adj. *R^2^* | .06 | .003 | .01 |
| Residual *SE* (df = 698) | .97 | 1.00 | .99 |
| *F* (1, 698) | 46.80^***^ | 2.83^†^ | 8.34^**^ |
|  | | | |
| *Note.* ^†^ *p* < .10, * *p* < .05, ** *p* < .01, *** *p* < .001. Number of observations = 700. Separate models predicting correlation coefficient (*r_τ_*) between the MFQ and the MFCT, and log RT and log *τ* on the MFCT. All variables have been standardised. *SE* is provided in parentheses. | | | |

# References

Ahluwalia, A. (2015). Mapping Moral Intuitions: Intuitive processing of moral foundations (MSc).

University of Edinburgh, Edinburgh. http://hdl.handle.net/1842/16708

Ahluwalia, A. (2020). The Moral Foundations Conflict Task: Measuring intuitive conflict between moral foundations using a novel task (PhD). University of Edinburgh, Edinburgh. doi:10.7488/era/1306

Eisinga, R., Grotenhuis, M., & Pelzer, B. (2013). The reliability of a two-item scale: Pearson, Cronbach, or Spearman-Brown? *International Journal of Public Health, 58*(4), 637-642. doi:10.1007/s00038-012-0416-3

Graham, J. (2010). *Left gut, right gut: Ideology and automatic moral reactions.* (PhD). University of Virginia, Charlottesville.

Graham, J., Haidt, J., & Nosek, B. A. (2009). Liberals and conservatives rely on different sets of moral foundations. *Journal of Personality and Social Psychology*, *96*(5), 1029-1046. doi:10.1037/a0015141

Heathcote, A., Popiel, S. J., & Mewhort, D. J. (1991). Analysis of response time distributions: An example using the Stroop task. *Psychological Bulletin, 109*(2), 340-347. doi:10.1037/0033-2909.109.2.340

Jeffreys, H. (1939/1961). *The Theory of Probability* (1st/3rd ed.). Oxford University Press.

Kaplan, R. M., & Saccuzzo, D. P. (2001). *Psychological Testing: Principles, Applications, and Issues* (5 ed.). Belmont, CA, USA: Wadsworth/Thomson Learning.

Lacouture, Y., & Cousineau, D. (2008). How to use MATLAB to fit the ex-Gaussian and other probability functions to a distribution of response times. *Tutorials in Quantitative Methods for Psychology, 4*(1), 35-45. doi:10.20982/tqmp.04.1.p035

Luce, R. D. (1986). Two-Choice Reaction Times: Basic Ideas and Data. In *Response Times: Their Role in Inferring Elementary Mental Organization* (pp. 205-271). Oxford University Press.

Massidda, D. (2013). retimes: Reaction Time Analysis (Version 0.1-2). Retrieved from https://CRAN.R-project.org/package=retimes

McGill, W. J. (Ed.) (1963). *Stochastic latency mechanisms* (Vol. 1). Wiley.

Moore, A. B., Lee, N., Clark, B., & Conway, A. (2011). In defense of the personal/impersonal distinction in moral psychology research: Cross-cultural validation of the dual process model of moral judgment. *Judgment and Decision Making, 6*(3), 186-195. doi:10.1017/S193029750000139X

Morey, R. D. (2008). Confidence Intervals from Normalized Data: A correction to Cousineau (2005). *Tutorials in Quantitative Methods for Psychology, 4*(2), 61-64. doi:10.20982/tqmp.04.2.p061

Van Berkel, L., Crandall, C. S., Eidelman, S., & Blanchar, J. C. (2015). Hierarchy, Dominance, and Deliberation: Egalitarian Values Require Mental Effort. P*ersonality and Social Psychology Bulletin*, *41*(9), 1207-1222. doi:10.1177/0146167215591961
